# Tumor suppressive miR-99b-5p as an epigenomic regulator mediating mTOR/AR/SMARCD1 signaling axis in aggressive prostate cancer

**DOI:** 10.3389/fonc.2023.1184186

**Published:** 2023-11-07

**Authors:** Mohammad Waseem, Himali Gujrati, Bi-Dar Wang

**Affiliations:** ^1^ Department of Pharmaceutical Sciences, University of Maryland Eastern Shore School of Pharmacy and Health Professions, Princess Anne, MD, United States; ^2^ Hormone Related Cancers Program, University of Maryland Greenebaum Comprehensive Cancer Center, Baltimore, MD, United States

**Keywords:** miR-99b-5p mimic, prostate cancer disparities, castration-resistant prostate cancer, mTOR/AR signaling, enzalutamide-resistant, androgen independent

## Abstract

**Introduction:**

African American (AA) men exhibited 2.3-fold higher PCa incidence and 1.7-fold higher PCa mortality rates when compared to the European American (EA) men. Besides the socioeconomic factors, emerging evidence has highlighted that biological risk factors may play critical roles in the AA PCa disparities. Previously, we have shown that downregulated miR-99b-5p and upregulated mTOR cooperatively promotes the AA PCa aggressiveness and drug resistance.

**Methods:**

In this study, we aimed to explore the miR-99b-5p/mTOR/AR/SMARCD1 signaling axis in AA PCa aggressiveness. The analyses used in the study included immunofluorescence, western blot, *in-vitro* functional assays (TUNEL, colony forming, and MTT), and chromatin immunoprecipitation (ChIP)-qPCR assays in 2D and/or 3D culture model of EA PCa and AA PCa cell lines.

**Results:**

Specifically, the immunofluorescence staining, and western blot analysis has revealed that nuclear mTOR, AR, and SMARCD1 were highly expressed in AA PCa (MDA PCa 2b) compared to EA PCa (LNCaP) cell line. Western blot analysis further revealed that miR-99b-5p inhibited protein levels of mTOR, AR/AR-V7 and SMARCD1 in cytoplasm and nuclei of EA and AA PCa. The in-vitro functional (MTT, TUNEL, and clonogenic) assays have demonstrated that miR-99b-5p effectively inhibited cell proliferation/survival and induced cell apoptosis in EA and AA PCa cells. Moreover, combination of miR-99b-5p and enzalutamide (Enz) synergistically enhances the cytotoxicity against aggressive AA PCa and castration-resistant prostate cancer (CRPC). mTOR ChIP-qPCR assays further demonstrated that miR-99b-5p or miR-99b-5p/Enz significantly reduces the recruitment of mTOR to the genes involved in the metabolic reprogramming in CRPC.

**Discussion:**

Taken together, miR-99b-5p may function as an epigenomic driver to modulate the mTOR/AR/SMARCD1 signaling axis in AA PCa and resistant CRPC.

## Introduction

Prostate cancer (PCa) has been considered as one of the leading cancer-associated mortalities among men in the globe. In the United States, PCa is the most frequently diagnosed cancer (268,490 estimated new cases in 2022) and the second leading cause of cancer deaths (34,500 estimated deaths in 2022) among men (1). Notably, African American (AA) men exhibit 2.3-time higher PCa incidence and 1.7-time higher PCa mortality rates, when compared to European American (EA) men (1). Besides the socioeconomic factors, multiple genetic/epigenetic factors have been associated with the PCa disparities observed between EA and AA PCa. Emergent evidence suggests that alteration in epigenetic/epigenomic mechanisms may play critical roles in promoting the AA PCa disparities. For instance, differential DNA methylation patterns (2–4) and deregulated microRNA (miRNA) regulatory networks (5–8) have been implicated in promoting the AA PCa disparities.

MiRNA are short non-coding RNAs regulating protein expression through degradation of target mRNAs or inhibition of protein translation. Emerging evidence has implicated miRNAs as potential biomarkers in cancers, including PCa (9–13). A correlation-based approach, combining miRNA and mRNA (or protein) profiling data with mRNA target prediction to identify the miRNAs and mRNA targets with inverse regulatory correlation, has proved to be a more effective way to identify critical miRNA-mRNA interactions in cancers (14–16). Our previous genomic study has identified dozens of the ‘reciprocal miRNA-mRNA pairings’ that are differentially expressed between AA PCa and EA PCa (6). These reciprocal miRNA-mRNA pairings demonstrated significant regulatory effects on activating multiple oncogenic pathways such as ERBB, mTOR, VEGF and HIF-1α signaling, in AA PCa (6, 17). Among these miRNA-mRNA pairing, tumor suppressive miR-99b-5p negatively regulates *MTOR* expression (at mRNA and protein levels) and play a central role in regulating PI3K/AKT/mTOR/HIF-1α/VEGF signaling axis (5). Our previous study further revealed that miR-99b-5p also targets/inhibits *AR* (encoding androgen receptor, AR) expression at protein level, consequently inhibiting AR/mTOR signaling and AR/mTOR translocation to the nucleus (17). Since nuclear AR and mTOR transcriptionally activate hundreds of genes in AA PCa and CRPC, miR-99b-5p potentially functions as an epigenomic driver mediating the AA PCa aggressiveness and drug resistance.

In PCa pathogenesis, androgen-receptor (AR) has been shown as a key player for early detection of the disease (18, 19). AR signaling axis play a critical functional role for normal male reproductive function, and is regulated in subsequent binding of androgens (such as dihydrotestosterone, DHT) to its specific receptor, causing its nuclear events followed by the regulation of transcriptional targets such as prostate-specific antigen (PSA) and transmembrane protease, serine 2 (TMPRSS2) (20). Androgen deprivation therapy (ADT) has been a systemic treatment of both localized and advanced PCa. Unfortunately, a considerable majority of ADT targeted patients ultimately progress to castration-resistant prostate cancer (CRPC) in a therapeutic window of 2–3 years (21, 22). CRPC has also been characterized by constant tumor growth despite the castrated levels of serum testosterone, ultimately leading to significant patient deaths. Several AR antagonists, such as enzalutamide (Enz, a nonsteroidal antiandrogen), and abiraterone acetate (an irreversible inhibitor of CYP17A1) were approved by FDA for therapeutic alternative for aggressive and metastatic PCa (23, 24). However, a group of patients have shown a resistance to both Enz and abiraterone due to the expression of oncogenic *AR* splice variants, such as *AR-V7* (25). AR is a 110-kDa transcription factor classified in the superfamily of steroid hormone receptor. The phosphoinositide 3-kinase (PI3K)-AKT-mTOR signaling pathway has clearly demonstrated as a core mechanism that regulates ADT resistance and triggers tumor growth at castrated levels of testosterone. Indeed, this regulatory pathway has been shown as alterations at both genomic and transcriptional level in almost all aggressive PCa (26). Therefore, cancer cells are actively utilizing this pathway to adapt to the cellular stress triggered by ADT. Furthermore, recent investigations have established a direct link between mTOR and AR signaling, conveying a progressive reciprocation on these molecular targets during the development of androgen insensitivity (27, 28). AR and mTOR have been confirmed as direct target of miR-99b-5p. Overexpression of miR-99b-5p inhibits AR, mTOR, and PSA expression levels, leading to inhibition of cell proliferation/migration, induction of autophagy and apoptosis (29). Additionally, it has been proposed that AR and mTOR co-activate a set of downstream genes involved in the metabolic reprogramming critical for CRPC progression (30). SMARCD1, a member of the SWI/SNF family proteins, is known as a cofactor of AR involved in activation of AR-target genes (31). Interestingly, miR-99b-5p also targets and negatively regulates SMARCD1 expression (32). Recent study has further demonstrated that SMARCD1 can regulated AR-target genes in androgen-dependent and -independent manners, suggesting its potential involvement in progression to CRPC (33). Taken together, miR-99b-5p (downregulated in AA PCa and CRPC) may play a pivotal role in regulating mTOR/AR/SMARCD1 signaling axis in aggressive PCa.

In this study, we aimed to study the functional involvements of the epigenomic driver, miR-99b-5p, in AA PCa disparities and the metabolic reprogramming in CRPC. A series of *in vitro* biochemistry, cellular and molecular biology, and functional assays were performed to elucidate the molecular mechanism of miR-99b/mTOR/AR/SMARCD1 signaling in AA PCa and CRPC. First, immunofluorescence assays were employed to explore the expression levels and subcellular distributions of mTOR, AR, AR-V7, and SMARCD1 in 2D (monolayer) and 3D (organoid) in PCa cell line models derived from EA PCa (LNCaP, PC-3, DU-145, and CRPC lines 22Rv1 and C4-2B) and AA PCa (MDA PCa 2b). Second, the EA and AA PCa cell lines were transfected with nonsense control RNA, miR-99b-5p mimic, Enz, or miR-99b-5p/Enz combination, then followed by immunofluorescence assays and western blot analysis. Specifically, immunofluorescence assays were used to visualize the protein expression and distribution under the four treatments, while the western blot assays were used to examine the protein levels of mTOR, AR, pAR and SMARCD1 in cytoplasmic and nuclear fractions of the EA and AA PCa cells under these treatments. Third, functional assays (MTT, colony forming/clonogenic, and TUNEL assays) were conducted in 2D and 3D cell cultures to examine the functional impacts of miR-99b-5p and Enz as single agents, and miR-99b-5p/Enz as combination therapy. Finally, chromatin immunoprecipitation-qPCR assays were used to investigate the binding affinity of mTOR/AR complex on their downstream target genes under the treatments. In summary, we have demonstrated the functional effects of miR-99b-5p and miR-99b-5p/Enz combination in suppressing EA PCa, CRPC and AA PCa. Our results suggest that miR-99b-5p-mediated mTOR/AR/SMARCD1 signaling may play a central role in promoting the aggressiveness and drug resistance in CRPC and AA PCa. Therefore, miR-99b-5p may serve as a precision prognostic biomarker and therapeutic tool for detecting and targeting the aggressive AA PCa and CRPC.

## Materials and methods

### Monolayer and organoid cell cultures

The human PCa cell lines PC-3, DU-145, LNCaP, 22Rv1, C4-2B and MDA PCa 2b were used in the current study. Specifically, PC-3 and DU-145 are AR-negative EA PCa cell lines derived from bone and brain metastasis, respectively. LNCaP represents an androgen-dependent EA PCa cell line derived from lymph node. 22Rv1 and C4-2B represent castration-resistant prostate cancer (CRPC) cell lines derived from EA PCa patients, while MDA PCa 2b represents an androgen-independent AA PCa cell lines derived from bone metastasis. All PCa cell lines, including LNCaP (ATCC CRL-1740), PC-3 (ATCC CRL-1435), DU145 (ATCC HTB-81), 22Rv1 (ATCC CRL-2505), C4-2B (ATCC CRL-3315) and MDA PCa 2b (ATCC CRL-2422), were purchased from American Type Culture Collection (ATCC, Manassas, VA, USA). LNCaP and 22Rv1 were cultured in RPMI-1640 with 10% fetal bovine serum (FBS). DU-145 and PC-3 were cultured in DMEM with 10% FBS. C4-2B was grown in advanced DMEM with 10% FBS, and MDA PCa 2b was cultured in BRFF-HPC1 with 20% FBS.

For establishing PCa organoids, the PCa cells (LNCaP, PC-3, DU-145, 22Rv1, C4-2B and MDA PCa 2b) were grown to 80% confluency, and washed three times with 1× PBS then treated with 0.25% trypsin-EDTA at 37°C. Thereafter, the cell suspension was centrifuged at 150×g at 4°C for 5 min, and resuspended in advanced DMEM/F12 media then mixed with pre-thawed matrigel (Corning Life Sciences, Tewksbury, MA, USA) and dropped at the middle of the well of a pre-warmed culture plate. Then, cell culture plates were placed at upside down in the incubator with 5% CO_2_ at 37°C for 15 min to allow the matrigel to solidify into a dome. After 15 min, the pre-warmed organoid medium (diluted B27, 1.25 mM N-acetyl-L-cysteine, 5 ng/ml EGF, 100 ng/ml Noggin, 500 ng/ml recombinant R-spondin 1, 500 nM A83-01, 10 ng/ml FGF10, 5 ng/ml FGF2, 1 µM PGE2, 10 mM nicotinamide, 10 µM SB202190, 1 nM DHT, and 10 µM Y-27632 dihydrochloride) was added and the cells were grown in 5% CO_2_ at 37°C for 14 days. These cell lines and organoids were served as an *in vitro* model to examine the functional action of miR-99b-5p/MTOR/AR pairing and Enz in both EA PCa and AA PCa cell lines and their derived organoid.

### MicroRNA transfection and drug treatment in EA and AA PCa cell models

To examine the inhibitory effects of miR-99b-5p and Enz treatments in EA and AA PCa, the EA PCa LNCaP, EA CRPC (22Rv1 and C4-2B) and AA PCa MDA PCa 2b cell lines were seeded at a density of 3 × 10^5^ cells/well in 6-well plates. The PCa cells (2D or 3D cultures) were grown for 24 h followed by the transfection with nonsense RNA or miR-99b-5p mimic (Ambion, Austin, TX, USA) using DharmaFECT4 transfection reagent (Dharmacon, Lafayette, CO, USA). After transfection for 24 h, the PCa cells were treated with either vehicle or enzalutamide for additional 48 h. The four treatment groups were designated as NC (nonsense control, referring to nonsense RNA with vehicle control), miR-99b-5p (miR-99b-5p mimic with vehicle), Enz (nonsense RNA with 20 μM enzalutamide), and miR-99b-5p/Enz (miR-99b-5p mimic with 20 μM enzalutamide). 20 μM of Enzalutamide concentration was used in the experiments, according to previous studies (34–36).

### Western blot analysis

The western blot assays were performed using standardized protocol established in our lab as previously described (5, 17, 37). Briefly, the PCa cells were collected and total proteins were extracted using M-PER Mammalian Protein Extraction Reagent (Thermo Fisher Scientific, Waltham, MA, USA) with protease and phosphatase inhibitor cocktail (Thermo Fisher Scientific, Waltham, MA, USA). Quantification of protein concentrations from individual protein samples were determined using BCA assay kit (Thermo Fisher Scientific, Waltham, MA, USA). Bolt 4-12% Bis-Tris mini protein gels (Thermo Fisher Scientific, Waltham, MA, USA) were used for running protein gel electrophoresis. The primary antibodies used in the study were rabbit monoclonal antibodies against AR/AR-V7 (catalog# 5153, 1:1000), pAR (catalog#16969, 1:1000), mTOR (catalog#2972, 1:1000), GAPDH (catalog#5174, 1:1000), and Lamin B1(catalog# 13435, 1:1000) from Cell Signaling Technology (Danvers, MA, USA), and polyclonal rabbit antibody against SMARCD1 from Invitrogen (catalog# PA5-30175, 1:1000, Waltham, MA, USA). The secondary antibody used were anti-rabbit IgG-HRP (catalog# 4030-05, 1:10000) and anti-mouse IgG-HRP (catalog# 1033-05, 1:10000) antibodies purchased from Thermo Fisher Scientific (Waltham, MA, USA).

### Immunofluorescence staining for 2D monolayer and 3D cultures

For this procedure, 4×10^4^ cells were seeded on cover slip and allowed to attach for 24 h in 5% CO_2_ incubator at 37°C. All the experimental cells were subjected to immunofluorescence assays 48 h following transfection of NC or miR-99b-5p mimic, and/or Enz treatment. Briefly, cells were washed with 1×PBS, fixed in 4% paraformaldehyde, and permeabilized with 0.1% Triton X-100. Cells were then blocked for 1 h with 2% BSA in 1×PBS. Primary antibodies against mTOR (catalog# 2972, Cell Signaling Technology, Waltham, MA, USA, or catalog# sc-517464, Santa Cruz Biotechnology, Dallas, TX, USA), AR (catalog# 5153, from Cell Signaling Technology, Waltham, MA, USA, or catalog# sc-7305, Santa Cruz Biotechnology, Dallas, TX, USA), SMARCD1 (catalog# PA5-30175, Invitrogen, Waltham, MA, USA) and were applied to the fixed/permeabilized cells for incubation overnight at 4 ◦C. The cells were washed twice with 1×PBS and followed by the incubation of Alexa-Fluor-488-conjugated anti-rabbit and Alexa-Fluor-594-conjugated anti-mouse antibodies, respectively (catalog# A32731 and #A32744, from Invitrogen, Waltham, MA, USA) for 1 h at room temperature. Thereafter, cells were washed twice with 1×PBS for 5 min and the nuclei were visualized by staining with DAPI from Invitrogen (catalog# P36981, Waltham, MA, USA). The fluorescence-labeled cells were mounted on glass slides and visualized by fluorescence microscopy (Olympus, Waltham, MA, USA). Cell images were captured from 3–4 random areas at 20× magnification by using CellSens V1.18 software (Olympus, Waltham, MA, USA).

For 3D immunofluorescence staining procedure: media was removed and the matrigel domes were washed with 1×PBS, fixed in 4% paraformaldehyde for 30-60 min at room temperature. After fixation, the matrigel was dissolved, and the domes were detached to release organoids from matrigel by using differential centrifugation process. The resulting pellet was then suspended in 1×PBS and the suspension containing organoids was spread on the cover slips followed by incubation for 15-20 min to evaporate the 1×PBS at 37°C. Thereafter, the organoids were permeabilized, blocked, labeled by primary antibodies and secondary antibodies conjugated with fluorescence, mounted in presence of DAPI, and visualized under fluorescence microscope using the same protocol described for the 2D cultures. Images were captured from 3–4 random areas at 20× magnification by using CellSens V1.18 software (Olympus, Waltham, MA, USA).

### TdT-mediated dUTP-biotin end-labeling assays

TUNEL assay was performed as per the manufacturer’s protocol (Click-iT™ Plus *In-situ* Apoptosis Detection with Alexa Fluor Dyes, Thermo Fisher Scientific, Waltham, MA, USA). Specifically, 4×10^4^ cells were seeded on coverslips and allowed to attach for 24 h in 5% CO_2_ incubator at 37°C. After treatment of NC RNA, miR-99b-5p mimic, and/or Enz for 48 h, cells were fixed in 4% paraformaldehyde and washed three times with 1×PBS followed by the incubation with 0.25% Triton X-100 in 1×PBS and conclusively the cells were incubated with differential steps of TUNEL reaction mixture as per the manufacturer’s protocol. At the end of the reaction, the processed cells were counterstained with DAPI for 5 min, at room temperature in the dark. All the fluorescence-labeled cells were mounted on glass slides and visualized by Olympus BX3 fluorescence microscope (Olympus, Waltham, MA, USA). Cell images were captured from 3–4 random areas at 10× magnification by using CellSens V1.18 software (Olympus, Waltham, MA, USA).

### Cell viability assays for PCa organoid cultures

After miRNA transfection and/or drug treatment regimen, organoid formation/growth was captured by brightfield or phase contrast microscopy. In addition, the cell viabilities of the PCa organoids were assessed by 3-(4,5−dimethylthiazol−2−yl)−2,5 diphenyl tetrazolium bromide (MTT) solution. Briefly, 0.5 mg/ml MTT was added to the organoid cultures and incubated at 37°C for 3 h. The matrigel containing the PCa organoids was solubilize in a 2% sodium dodecyl sulfate solution for 2 h. Thereafter, 100 μl DMSO was added for 1 h to solubilize the reduced MTT or tetrazolium salt crystals, and the absorbance at the wavelength of 570 nm was detected using Mutiskan FC microplate photometer (Thermo Fisher Scientific Waltham, MA, USA). The cell viabilities of the organoids were presented as percentages of absorbance values from the controls. The data were analyzed using the Prism 9 program (GraphPad Software, La Jolla, CA) for graphing and statistical analysis.

### Colony forming (clonogenic) assay

The EA PCa LNCaP, EA CRPC (22Rv1 and C4-2B), and AA PCa (MDA PCa 2b) cells were seeded at a density of 500 cells/well in triplicate formats. After 24 h, cells were transfected and/or treated for 24 h and kept at 37°C in 5% CO_2_ for 14 days. The media was replaced every 3 days. At the end of experiment, cells were fixed with 90% chilled methanol followed by staining with 0.5% crystal violet. The colonies were counted and scored as percentage of the control experimental group.

### Chromatin immunoprecipitation assay

First, the PCa cell lines LNCaP, 22Rv1, C4-2B, and MDA PCa 2b were treated with nonsense RNA, miR-99b-5p, and/or Enz. After 48 h, the cells were harvested and subjected to ChIP assays using ChIP assay kit (Millipore, Burlington, MA, USA) according to the manufacture’s protocol. Briefly, 1×10^6^ cells were treated with 1% formaldehyde for 15-20 min at room temperature, to crosslink the proteins with DNA. After that, the cells were washed twice with 1×PBS, collected by scraping cells from the plates, then resuspended in SDS lysis buffer for 10 min on ice. The cell lysates were sonicated for shearing DNA to lengths between 250 and 750 bp, then the lysates were centrifuged at 1,4000×g for 20 min at 4°C. Thereafter, the anti-mTOR antibody was added to immunoprecipitate the mTOR/DNA complexes at 4°C for 1 h with agitation. 50 μl of protein A agarose/salmon sperm DNA slurry was then added for incubation at 4°C overnight. After incubation, the solution was spun down and the agarose/antibody/protein-DNA complexes were washed by low salt, high salt and LiCl immune complex wash buffers, and TE buffer, respectively. The agarose/antibody/protein-DNA complexes were then added with 8μl of 5M NaCl and incubated at 65°C for 8 h to reverse the DNA/protein crosslinks. The Input and ChIP samples were then subjected to qPCR reactions to check the mTOR occupancies (determined by the ratios of mTOR ChIP/total Input) on *KLK3, ENO1, SLC26A3* and *TMPRSS2* genes. The primer sequences were listed as follows: *KLK3*-forward: 5’-TGCCACTGGTGAGAAACCTGAGAT-3’, *KLK3*-reverse: 5’-TCAGAGACAAAGGCTGAGCAGGTT-3’, *ENO1*-forward: 5’-GGGGCTTATGTCTTGCCAGT-3’, *ENO1*-reverse: 5’-AGAGGTTTCCATTGGTTACTTGGT-3’, *SLC26A3*-forward: 5’-AGGATGTGGGCATCTTTGGG-3’, *SLC26A3*-reverse: 5’-GAGATAGCAGCCAGGCACAA-3’, *TMPRSS2*-forward: TGAGACTAGCCTGGACACCA-3’, and *TMPRSS2*-reverse: 5’-AAGCTCACTGCAGCCTCAAA-3’.

### Statistical analysis

Results were expressed as mean ± standard deviation (SD). All data were analyzed by using analysis of variance (ANOVA) followed by Tukey’s test. Values of p < 0.05 were considered as significant. All the statistical analyses were performed using GraphPad Prism 9.0 (Graph Pad Software, Inc., San Diego, CA, USA).

## Results

### Subcellular localizations of AR, AR-V7, mTOR and SMARCD1 in EA and AA PCa cells

**Figure 1:**
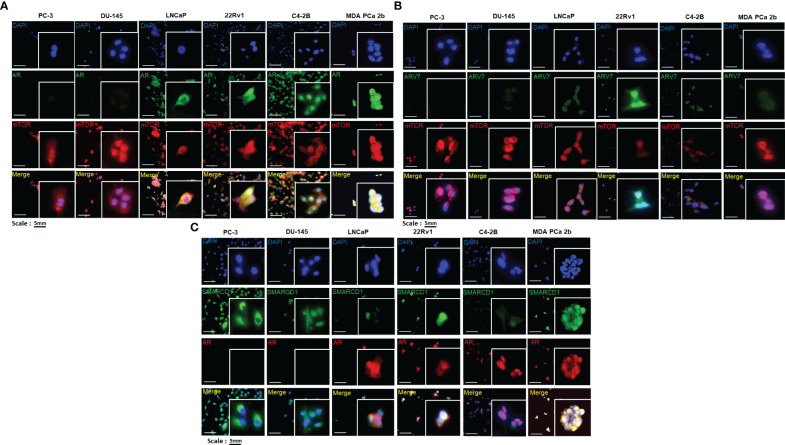
Immunofluorescence staining revealed the cellular localizations and expression levels of AR, AR-V7, mTOR, and SMARCD1 in EA PCa and AA PCa cell lines. **(A)** Immunofluorescence staining showed mTOR (red fluorescence) and AR (green fluorescence) signals in EA PCa cell lines (22Rv1, LNCaP, C4-2B, PC-3 and DU-145), and AA PCa cell lines (MDA PCa 2b). Nuclei were visualized by counterstaining with DAPI (blue fluorescence). Merged images were acquired by imposing DAPI, mTOR and AR signals to depict whether colocalization (yellow) of both mTOR and AR was observed either in nuclei or cytoplasm. **(B)** Immunofluorescence demonstrating AR-V7 (green fluorescence) and mTOR (red fluorescence) signals in EA PCa (22Rv1, LNCaP, C4-2B, PC-3 and DU-145) and AA PCa cell lines (MDA PCa 2b). Nuclei were visualized by counterstaining with DAPI (blue fluorescence). Merged images were achieved by overlaying DAPI, mTOR and AR-V7 signals to depict the colocalization (yellow) of both AR-V7 and mTOR either in nuclei or cytoplasm. **(C)** Immunofluorescence depicting SMARCD1 (green fluorescence) and AR (red fluorescence) signals in EA PCa cell lines (22Rv1, LNCaP, C4-2B, PC-3 and DU-145), and AA PCa cell lines (MDA PCa 2b). Merged images were achieved by overlaying DAPI, SMARCD-1 and AR signals to depict the colocalization (yellow) of both SMARCD-1 and AR either in nuclei or cytoplasm. All the captured fluorescent images were analyzed by using CellScans software V1.18. Five to six random images were captured by using 200× magnification.

To understand the regulation of miR-99b-5p/mTOR/AR signaling, the cellular locations of AR, AR-V7, mTOR and SMARCD1 were explored in EA PCa (LNCaP, PC-3, DU-145, 22Rv1, and C4-2B) and AA PCa (MDA PCa 2b) cell lines. LNCaP is an androgen-sensitive PCa, PC-3 and DU-145 are AR-negative cell lines derived from bone and brain metastases, while C4-2B (derived from parental line LNCaP) and 22Rv1 represented CRPC cell lines from EA. MDA PCa 2b is an AR-mutated and androgen-independent AA PCa derived from the bone metastasis. The immunofluorescence results confirmed that there was no AR expression in PC-3 or DU-145, whereas an abundant AR expression level was observed in LNCaP, 22Rv1, C4-2B and MDA PCa 2b (green fluorescence signals in **Figure 1A**). Notably, CRPC 22Rv1, C4-2B and AA PCa MDA PCa 2b demonstrated significantly higher expression levels of nuclear AR, compared to LNCaP that predominately expressed cytoplasmic AR (green fluorescence and merged images in **Figure 1A**). In contrast, mTOR protein was generally expressed in cytoplasm and nuclei of all the EA and AA PCa cell lines (red fluorescence signals in **Figure 1A**). The EA CRPC line, 22Rv1, particularly expressed a high level of nuclear AR-V7 splice isoform (green fluorescence signals in **Figure 1B**). We further examined the expression profiles of SMARCD1, an AR coactivator, in these six PCa cell lines. The immunofluorescence assays have revealed that SMARCD1 was not only expressed in cytoplasm of all PCa cell lines, but was expressed in the nuclear fractions in EA CRPC line 22Rv1 and AA PCa line MDA PCa 2b (green fluorescence in **Figure 1C**).

**Figure 2:**
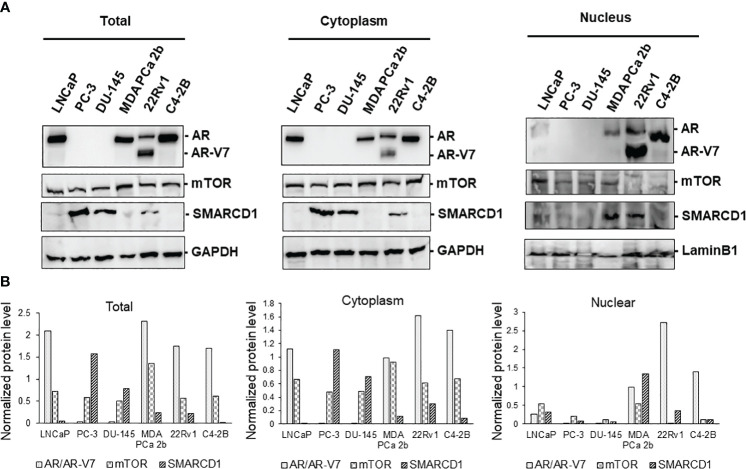
Western blot analyses of proteins expression levels of AR, AR-V7, mTOR, and SMARCD1 in EA PCa (22Rv1, LNCaP, C4-2B, PC-3 and DU-145) and AA PCa (MDA PCa 2b) cell lines. **(A)** Representative western blot images and **(B)** quantification of AR, AR-V7, mTOR, and SMARCD1 protein levels from total cell lysates (Total), cytoplasmic lysates (Cytoplasm), and nuclear lysates (Nucleus) of the EA and AA PCa cells were presented. Total lysate, cytoplasmic and nuclear protein fractions were prepared and subjected to the western blot assays (n=3) of the protein indicated above. GAPDH and Lamin B1 were used as endogenous controls for cytoplasmic and nuclear proteins, respectively. The normalized protein level was determined by normalization of the intensity of AR/AR-V7, mTOR or SMARCD1 to the intensity of its corresponding control (GAPDH for Total or Cytoplasm, or Lamin B1 for Nucleus).

Next, western blot assays were conducted to verify the total, cytoplasmic and nuclear protein levels of AR, AR-V7, mTOR, and SMARCD1 in these six PCa cell lines. Consistent with the expression patterns shown in the immunofluorescence assays, AR was expressed in LNCaP, 22Rv1, C4-2B and MDA PCa 2b, mTOR was comparably expressed in all PCa cell lines, and differential expression levels of SMARCD1 were observed in different cell lines (**Figure 2**). Interestingly, nuclear AR/AR-V7 and/or nuclear SMARCD1 were expressed in either EA CRPC (22Rv1, C4-2B) or AA PCa (MDA PCa 2b). Nuclear mTOR, a more oncogenic form of mTOR, was highly expressed in MDA PCa 2b cells (**Figure 2**). Taken together, nuclear AR/AR-V7, SMARCD1 and mTOR (i.e. active/phosphorylated forms of proteins) were enriched in androgen-independent AA PCa (MDA PCA 2b), and nuclear AR/AR-V7 and SMARCD1 were enriched in EA CRPC (22Rv1 and C4-2B), potentially explaining the aggressiveness of AA PCa and CRPC.

**Figure 3:**
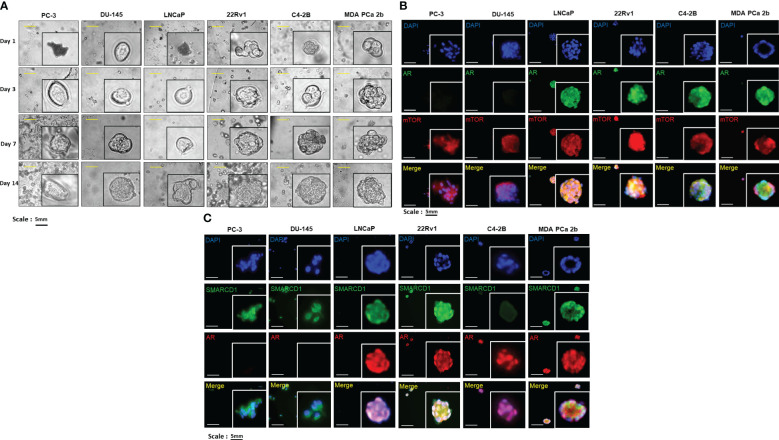
Bright-field imaging and immunofluorescence staining for 3D cultures derived from EA and AA PCa cell lines. **(A)** Morphological changes of organoids derived from EA PCa (PC-3, DU-145, LNCaP, 22Rv1, and C4-2B) and AA PCa (MDA PCa 2b), at different time points up to 14 days. **(B)** Immunofluorescence showing mTOR (red fluorescence) and AR (green fluorescence) signals in 3D cultures developed from EA PCa (22Rv1, LNCaP, C4-2B, PC-3 and DU-145) and AA PCa (MDA PCa 2b) cell lines. Nuclei were visualized by counterstaining with DAPI (blue fluorescence). Merged images were acquired by imposing DAPI, mTOR and AR signals to evaluate the colocalization (yellow) of mTOR and AR in nuclei or cytoplasm. **(C)** Immunofluorescence showing AR (red fluorescence) and SMARCD1 (green fluorescence) signals in organoids developed from EA PCa (22Rv1, LNCaP, C4-2B, PC-3 and DU-145) and AA PCa (MDA PCa 2b) cell lines. Nuclei were visualized by counterstaining with DAPI (blue fluorescence). Merged images were acquired by overlapping DAPI, AR and SMARCD1 signals to visualize the colocalization (yellow) of AR and SMARCD1 either in nuclei or cytoplasm. All the captured fluorescent images were analyzed by using CellScans software V1.18. Five to six random images were captured by using 200× magnification.

To further examine the expression levels/patterns and functional implications of AR/AR-V7, mTOR and SMARCD1 in consideration of tumor microenvironment, the 3D cultures were developed from PC-3, DU-145, LNCaP, 22Rv1, C4-2B and MDA PCa 2b cells. As shown in **Figure 3A**, all the cell lines were grown in the matrigels and the volumes of the PCa organoids were consistently increased in a time dependent manner. At the initial stage of organoid culture (day 1), all the experimental PCa cells prominently exhibited mono- or bi-layer growing patterns in matrigels, with 2-4 cells aggregated together. On day 3 and 7, organoid cultures were gradually formed, evident from the aggregation of dozens of cells. On day 14, the organoids of the PCa cells were established, and the organoids were subjected to immunofluorescence assays for visualizing the expression levels/patterns of AR/AR-V7, mTOR, SMARCD1. Similar to the expression profiles shown in the 2D (mono layer) cultures, AR was not expressed in PC-3 and DU-145 but highly expressed in LNCaP, 22Rv1, C4-2B and MDA PCa 2b organoids (green fluorescence, **Figure 3B**). Notably, higher nuclear AR levels were observed in EA CRPC (22Rv1 and C4-2B) and AA PCa (MDA PCa 2b) organoids (**Figures 3B, C**). The AR co-activator SMARCD1 was predominately expressed in cytoplasm of PC-3 and DU-145. In contrast, nuclear SMARCD1 was particularly enriched in EA CRPC 22Rv1 and AA PCa MDA PCa 2b cells (**Figure 3C**). These results, again, showed a generalized consistency with the results from western blot assays (**Figure 2**).

### Transfection of miR-99b-5p mimic and treatment of Enz inhibit the expression levels and/or nuclear translocation of mTOR, SMARCD1 and AR in EA and AA PCa Cells

**Figure 4:**
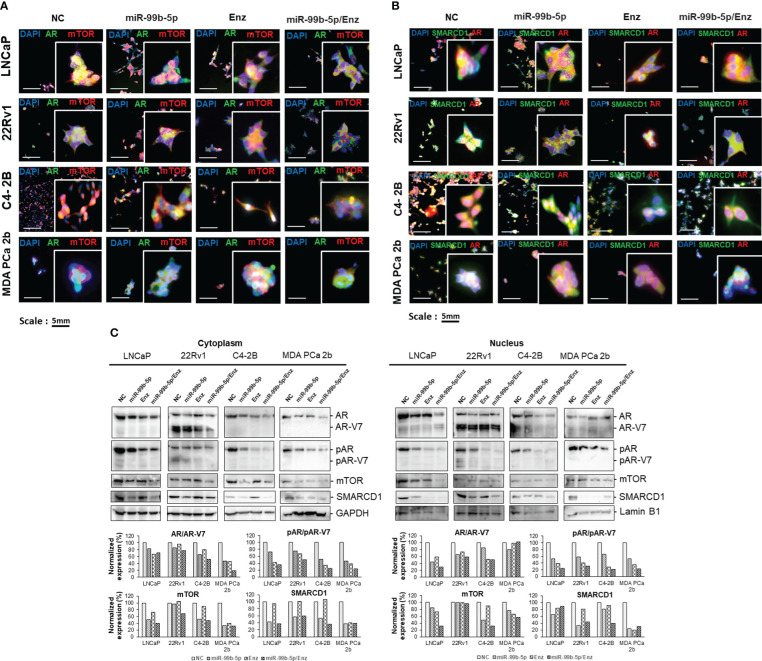
Immunofluorescence staining and western blot assays of the EA PCa and AA PCa cells treated with nonsense control (NC), miR-99b-5p mimic, Enz, or miR-99b-5p/Enz combination. **(A)** Immunofluorescence showing mTOR (red fluorescence) and AR (green fluorescence) signals in EA PCa (22Rv1, LNCaP, C4-2B) and AA PCa (MDA PCa 2b) cell lines under treatments. Merged images were presented by overlaying AR/AR-V7 (green fluorescence), mTOR (red fluorescence) and DAPI (blue fluorescence) signals to visualize their cellular locations. **(B)** Immunofluorescence assays to examine the SMARCD1 (green fluorescence) and AR (red fluorescence) signals in EA PCa (22Rv1, LNCaP, C4-2B) and AA PCa (MDA PCa 2b) cell lines. The green and red fluorescence signals overlapped with blue fluorescence signal (DAPI) to visualize the cellular distribution of AR and SMARCD1 in EA and AA PCa cells under treatments. All the captured fluorescent images were analyzed by using CellScans software V1.18. Five to six random images were captured by using 200× magnification. **(C)** Western blot analysis of mTOR, pAR, AR/AR-V7 and SMARCD1 protein levels in cytoplasmic and nuclear fractions. GAPDH and Lamin B1 were used as endogenous controls for cytoplasmic and nuclear proteins, respectively. The cells were cultured followed by transfection with nonsense RNA or miR-99b-5p mimic. 24 h after the transfections, the PCa cells were treated with vehicle, or Enz (20µM) for additional 48 h. NC, nonsense siRNA with vehicle control; miR-99b-5p, miR-99b-5p mimic with vehicle; Enz, nonsense RNA with enzalutamide; miR-99b-5p/Enz, miR-99b-5p mimic with enzalutamide.

To evaluate the inhibitory effects of miR-99b-5p and Enz on the protein levels and cellular locations of AR/AR-V7, mTOR and SMARCD1, immunofluorescence and western blot assays were performed in the AR-positive lines, including androgen-dependent EA PCa (LNCaP), EA CRPC (22Rv1, C4-2B), and AA PCa (MDA PCa 2b) cells in the absence and/or presence of miR-99b-5p and/or Enz. Specifically, the PCa cell lines were transfected/treated with nonsense/scrambled RNA, miR-99b-5p, 20 μM of Enz, or miR-99b-5p/Enz combination for 48 hr. As shown in **Figure 4A**, transfection of miR-99b-5p mimic caused a generalized reduction of mTOR (red fluorescence) and AR (green fluorescence) protein levels, compared to the NC, in all the four PCa cell lines. In contrast, no reduction of mTOR and AR signals was observed in any of the PCa cell lines when treated with Enz. These results reflected the function of Enz as an AR antagonist, which is not regulating the protein level of AR. Intriguingly, almost exclusive cytoplasmic mTOR and AR signals were detected in PCa cells under treatment of either miR-99b-5p, Enz, or miR-99b-5p/Enz combination (**Figure 4A**), suggesting both miR-99b-5p and Enz treatments block the nuclear translocation of AR and mTOR. Likewise, transfection of miR-99b-5p mimic decreased the protein levels of SMARCD1 (green fluorescence, **Figure 4B**) in LNCaP, 22Rv1, C4-2B and MDA PCa 2b cells. Also, either treatment of miR-99b-5p, Enz, or miR-99b-5p/Enz combination sequestered SMARCD1 in cytoplasm and the nuclear translocation of SMARCD1 was blocked (green fluorescence and merged images, **Figure 4B**). Taken together, our results suggested that miR-99b-5p targets/suppresses protein expression of AR, mTOR and SMARCD1, while both miR-99b-5p and Enz inhibit nuclear translocation of AR, mTOR and SMARCD1. Additionally, the full panels of immunofluorescence staining images for AR, mTOR, and SMARCD1 in all PCa cell lines under different treatments were shown in **Supplementary Figures 1-8**.

### Differential protein levels of AR/AR-V7, pAR/pAR-v7, mTOR, and SMARCD1 in cytoplasm and nuclei of EA and AA PCa cells in the presence/absence of miR-99b-5p mimic and/or Enz

To verify the cellular locations of AR, mTOR, and SMARCD1 under the treatments, western blot analyses were performed using the cytoplasmic and nuclear lysates from EA PCa (LNCaP, 22Rv1 and C4-2B) and AA PCa (MDA PCa 2b) cells grown in the androgen-containing media with the presence/absence of miR-99b-5p and/or Enz. As shown in **Figure 4C**, AR, mTOR and SMARCD1 levels were decreased in both cytoplasmic and nuclear fractions in LNCaP, C4-2B, and MDA PCa 2b cells treated with miR-99b-5p mimic vs. NC. Whereas, the western blot results revealed that slight to no reduction in cytoplasmic AR and mTOR signals and nuclear mTOR were detected in the 22Rv1 (EA CRPC) cells in response to miR-99b-5p. Notably, remarked decrease in cytoplasmic and nuclear AR/AR-V7, mTOR and SMARCD1 levels were observed when PCa cells were treated with combination of miR-99b-5p and Enz (**Figure 4C**). Phosphorylation states of cytoplasmic and nuclear AR/AR-V7 were reduced in all the PCa cells treated with miR-99b-5p.

In the presence of 20 μM Enz, phosphorylation states of cytoplasmic/nuclear AR were significantly decreased in the Enz-responding LNCaP, C4-2B and MDA PCA 2b cells, but not in Enz-resistant 22Rv1 cells (particularly pAR level in cytoplasm, in **Figure 4C**). Moreover, a synergistic inhibition of pAR was observed in all PCa cell lines (including 22Rv1) under the combined miR-99b-5p/Enz treatment (miR-99b-5p + Enz groups, in **Figure 4C**). These results strongly suggested that miR-99b-5p and Enz can effectively inhibit EA and AA PCa, and miR-99b-5p mimic can further sensitize the CRCP cell line 22Rv1 to Enz. Taken together, miR-99b-5p/Enz combined treatment could potentially serve as a novel therapy for Enz-resistant CRCP and androgen-independent AA PCa. Note that the phosphorylation states of cytoplasmic/nuclear AR were shown as basal levels in all treatment groups across four cell lines in the absence of androgen (pAR/AR ratios in **Supplementary Figure 9**, top panels). In the presence of androgens, cytoplasmic/nuclear AR was highly phosphorylated in NC treated cells. However, pAR states were reduced under treatment of miR-99b-5p, Enz, or miR-99b-5p/Enz combination (pAR/AR ratios in **Supplementary Figure 9**, bottom panels).

### Overexpression of miR-99b-5p induces cell apoptosis and diminishes cell viability in PCa cell lines and organoid model

**Figure 5:**
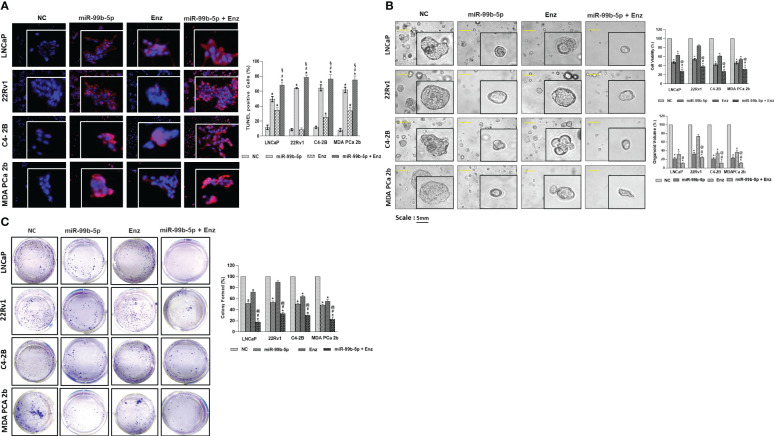
*In-vitro* functional assays (TUNEL, MTT, and clonogenic assays) were performed in EA PCa (22Rv1, LNCaP, C4-2B) and AA PCa (MDA PCa 2b) cell lines treated with NC or miR-99b-5p mimic in presence or absence of 20 μM of Enz. **(A)** TUNEL assays were carried out to visualize the DNA damages occurred during apoptotic events in PCa cell lines upon NC, miR-99b-5p, Enz, or miR-99b-5p/Enz treatments. Apoptotic events were detected based on the DNA damages (visualized as red fluorescent spots, defined as TUNEL-positive cells) in the nuclei (blue, DAPI staining). The red/purple signals derived from overlaying DAPI and TUNEL signals indicated the active apoptotic activities (DNA damages occurring in the nuclei) in the EA and AA PCa cells. For TUNEL, 3-4 random images were captured by using 100× magnification. Significantly different cell apoptotic capacities in miR-99b-5p vs. NC, Enz vs. NC or miR-99b-5p/Enz vs. NC (*p-value < 0.05) were determined based on ANOVA with Dunnett’s *post-hoc* test. Significantly different cell apoptotic capacities in miR-99b-5p/Enz vs. miR-99b-5p (^#^ *p*-value < 0.05) and miR-99b-5p/Enz vs. Enz (^§^ *p*-value < 0.05) were determined based on ANOVA with Tukey’s *post-hoc* test. Each value was represented as mean ± SD (*n* = 3). **(B)** Bright-field imaging and MTT assays of the EA (LNCaP, 22Rv1 and C4-2B) and AA (MDa PCa 2b) PCa organoids in response to NC, miR-99b-5p, Enz or miR-99b-5p/Enz treatments. Significantly different cell viabilities in miR-99b-5p vs. NC, Enz vs. NC or miR-99b-5p/Enz vs. NC (* *p*-value < 0.05) were determined by MTT assays based on ANOVA with Dunnett’s *post-hoc* test. Significantly different cell viability in miR-99b-5p/Enz vs. miR-99b-5p (^#^ *p*-value < 0.05) and miR-99b-5p/Enz vs. Enz (^§^ *p*-value < 0.05) were determined based on ANOVA with Tukey’s *post-hoc* test. Each value was determined by percentage of NC and each data point was represented as mean ± SD (*n* = 3-4). The volumes of organoids were calculated based on the equation: V = 4/3πR^3^, where V is volume and R is the radius averaged from 3-4 organoids. The volume of the NC treated organoid in each cell line was defined as 100%. Therefore, the relative organoid volume under treatment was determined by normalizing to its control (volume of miR99b-5p, Enz, or miR-99b-5p/Enz-treated organoid/volume of NC-treated organoid × 100%). Significantly different organoid volume in miR-99b-5p/Enz vs. miR-99b-5p (^#^ *p*-value < 0.05) and miR-99b-5p/Enz vs. Enz (^§^ *p*-value < 0.05) were determined based on ANOVA with Tukey’s *post-hoc* test. Each value was represented as mean ± SD (n = 3-4). **(C)** PCa colonies were counted and analyzed to evaluate the inhibitory effects by NC, miR-99b-5p, Enz or miR-99b-5p/Enz treatments. Significantly different colony forming capacities (*p-value < 0.05, in miR-99b-5p vs. NC, Enz vs. NC, or miR-99b-5p/Enz vs. NC) were determined based on ANOVA with Dunnett’s *post-hoc* test. Significantly different colony forming capacities in miR-99b-5p/Enz vs. miR-99b-5p (^#^ *p*-value < 0.05) and miR-99b-5p/Enz vs. Enz (^@^ *p*-value < 0.05) were determined based on ANOVA with Tukey’s *post-hoc* test. Each value (% of NC-treated group) was represented as mean ± SD (*n* = 3-4). The 2D and 3D culture were grown followed by transfection with nonsense RNA or miR-99b-5p mimic. 24 h after the transfections, the PCa cells were treated with vehicle, or Enz (20µM) for additional 48 h. NC, nonsense siRNA with vehicle control; miR-99b-5p, miR-99b-5p mimic with vehicle; Enz, nonsense RNA with enzalutamide; miR-99b-5p/Enz, miR-99b-5p mimic with enzalutamide.

To further assess the inhibitory effects of miR-99b-5p and/or Enz treatments in EA CRCP and AA PCa cells, *in-vitro* functional assays (TUNEL, MTT, colony forming assays) were conducted in 2D (monolayer) or 3D (organoid) PCa cultures treated with NC, miR-99b-5p, Enz, and miR-99b-5p/Enz combination. As shown in **Figure 5A**, TUNEL assay results have demonstrated enhanced DNA breakages (i.e., increased red fluorescent signals in nuclei) in the miR-99b-5p mimic vs. NC transfected in LNCaP, 22Rv1, C4-2B and MDA PCa 2b. In addition, Enz treatments significantly enhanced DNA breakage/cell apoptosis (i.e. increased red fluorescent signals in nuclei, accounting for 50-65% of total cells) in all EA and AA PCa cells, except the resistant 22Rv1 cells (**Figure 5A**). Notably, a drastically synergistic effect of apoptotic induction was observed across all the EA and AA PCa cell lines, including the Enz-resistant 22Rv1 (i.e. evident from the 70-80% of TUNEL-positive cells in all PCa cell lines under combination treatment, **Figure 5A**).

The cell viabilities were assessed by MTT assays in the organoid cultures developed from LNCaP, 22Rv1, C4-2B, and MDA PCa 2b under treatments of NC, miR-99b-5p mimic, Enz and/or miR-99b-5p/Enz combination. Specifically, transfections of miR-99b-5p mimic resulted in significant reductions (i.e. 40-55% decrease) of cell viabilities in all PCa organoids. On the other hand, the Enz treatments resulted in moderate reduction (40-45% decrease) of cell viabilities in LNCaP, C4-2B and MDA PCa 2b cells, while a slight decrease (~10% reduction) in cell viability was observed in 22Rv1 organoid culture (**Figure 5B**, right top panel). The miR-99b-5p/Enz combination, again, demonstrated a synergistic effect on inhibiting cell growth (ranging from 60-70% decrease in cell viabilities) in all the PCa organoids, including the Enz-resistant 22Rv1 organoid (miR-99b-5p + Enz groups, **Figure 5B**, right top panel). In contrast, transfection of miR-99b-5p inhibitor resulted in increased cell viabilities in all EA and AA PCa cell lines (miR-99b-5p inhibitor vs. NC, and miR-99b-5p inhibitor/Enz vs. Enz, **Supplementary Figure 10**). These results, again, have confirmed the synergistic inhibitory effect of cell growth by treating the EA CRPC and AA PCa with miR-99b-5p mimic/Enz combination therapy. Consistent with the cell viability results, a significant reduction in organoid volumes was observed in all the EA and AA PCa treated with miR-99b-5p or Enz. Furthermore, a significantly synergistic reduction of organoid volumes was observed in response to miR-99b-5p/Enz combined therapy in all EA and AA PCa organoids (**Figure 5B**, right bottom panel).

Next, the clonogenic (colony forming) assays were employed to examine the inhibitory effects of miR-99b-5p and/or Enz on LNCaP, 22Rv1, C4-2B, and MDA PCa 2b. Compared to the negative control (nonsense RNA/vehicle control), transfection of miR-99b-5p mimic significantly decreased the cell densities in all the EA and AA PCa cell lines. On the other hand, a significant suppression of cell growths (with 35-50% reduction of cell densities) was observed in Enz-responding LNCaP, C4-2B and MDA PCa 2b treated with Enz vs. NC. As anticipated, 22Rv1 has demonstrated a resistance to Enz (with only 10% decrease in cell density compared to NC, **Figure 5C**). The combination of miR-99b-5p (inhibiting AR/AR-V7, mTOR and SMARCD1 levels and their nuclear translocations) and Enz (inhibiting AR/AR-V7 activation) created synergistic effects on suppressing colony forming (with 70-85% decrease in cell densities) in all EA and AA PCa cell lines, including Enz-resistant 22Rv1 (**Figure 5C**). Morphological changes in cell shapes and nuclei were observed in EA and AA PCa cells treated with miR-99b-5p, Enz, or miR-99b-5p/Enz (**Supplementary Figure 11**), indicating that the treatments might cause cell damage/injury and induce cell apoptosis.

In summary, all the TUNEL, MTT and clonogenic assays have shown consistent results: 1) miR-99b-5p enhances cell apoptosis and inhibits cell viability/growth; 2) Enz effectively induces cell apoptosis and suppresses cell viability/survival in EA and AA PCa, except the Enz-resistant CRPC); 3) miR-99b-5p/Enz combination drastically enhances the cytotoxicity and inhibits cell growth in EA PCa, Enz-sensitive or resistant CRPC, and AA PCa. These results, again, strongly implicate a synergistic inhibitory effect is induced when combining miR-99b-5p with Enz for PCa treatment. Particularly, miR-99b-5p sensitizes CRPC and AA PCa to AR antagonist such as Enz.

### mTOR ChIP-qPCR assays revealed that miR-99b-5p mimic and Enz modulate the recruitment of AR/mTOR complex to its target genes

**Figure 6:**
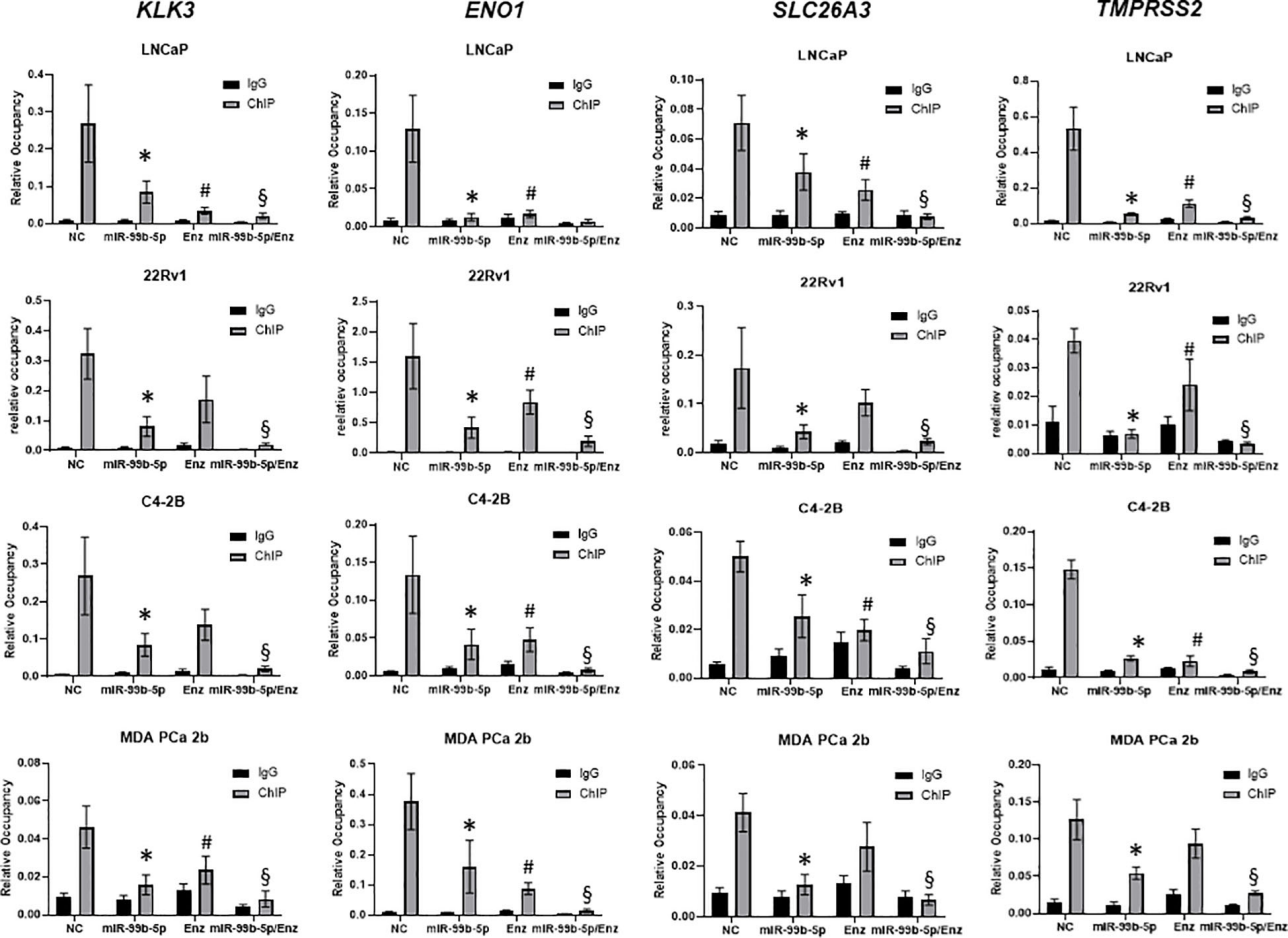
ChIP-qPCR assays for examining the binding affinities of mTOR to the mTOR target genes in EA and AA PCa cells under treatments of NC, miR-99b-5p, Enz, and miR-99b-5p/Enz. The mTOR ChIP assays were followed by qPCR assays of *KLK3, ENO1, SLC26A3*, and *TMPRSS2* (genes co-targeted by mTOR and AR). Significant differences of mTOR occupancies (*p-value < 0.05 in miR-99b-5p vs. NC, and ^#^ *p*-value < 0.05 in Enz vs. NC) on the target genes were determined based on ANOVA with Dunnett’s *post-hoc* test. Whereas, significant differences of mTOR occupancies in miR-99b-5p/Enz vs. miR-99b-5p and miR-99b-5p/Enz vs. Enz (^§^ *p*-value < 0.05) on the target genes were determined based on ANOVA with Tukey’s *post-hoc* test. Each value was determined by the ratio of ChIP signal/Input signal in qPCR reactions, and each data was derived from mean ± SD (*n* = 4). The PCa cells were cultured followed by transfection with nonsense RNA or miR-99b-5p mimic. 24 h after the transfections, the PCa cells were treated with vehicle, or Enz (20µM) for additional 48 h. NC, nonsense siRNA with vehicle control; miR-99b-5p, miR-99b-5p mimic with vehicle; Enz, nonsense RNA with enzalutamide; miR-99b-5p/Enz, miR-99b-5p mimic with enzalutamide.

Previous study has shown that mTOR forms complex with AR and the nuclear mTOR regulates the metabolic gene transcription/reprogramming in CRPC. We hypothesized that miR-99b-5 (that negatively regulates mTOR and AR expression and nuclear translocation) plays a crucial role in regulating metabolic reprogramming in CRPC. Therefore, overexpression of miR-99b-5p theoretically disrupts the recruitment of nuclear mTOR and nuclear mTOR/AR complex onto their target genes, consequently inhibiting the expression of metabolic genes in CRPC. To validate this hypothesis, mTOR ChIP assays were performed to immunoprecipitate the mTOR/DNA and mTOR-AR/DNA complexes from LNCaP, 22Rv1, C4-2B and MDA PCa 2b cells treated with NC, miR-99b-5p mimic, Enz, or miR-99b-5p/Enz combination. After mTOR ChIP assays, qPCR assays were conducted to examine the expression levels of the mTOR target genes including *KLK3, ENO1, SLC26A3* and *TMPRSS2.* Specifically, *KLK3* encodes PSA, *ENO1* and *SLC26A3* are metabolic genes actively involved in CRPC, and *TMPRSS2* is an androgen-driven gene promoting PCa progression/metastasis. According to the study from Audet-Walsh et al., *ENO1* and *TMPRSS2* were identified as mTOR target genes, and *KLK3* and *SLC26A3* were identified as mTOR/AR co-target genes (30). As anticipated, the mTOR ChIP-qPCR assays have revealed that mTOR (or mTOR/AR) was enriched on *KLK3, ENO1, SLC26A3* and *TMPRSS2* genes in LNCaP, 22Rv1, C4-2B, and MDA PCa 2b (NC treatment, **Figure 6**). In contrast, miR-99b-5p mimic significantly reduced the mTOR occupancies on these four genes in all cell lines, including EA PCa, CRPC and AA PCa cell lines (miR-99b-5p vs. NC, **Figure 6**). Whereas, Enz treatments significantly reduced the recruitment of mTOR (or mTOR/AR) to *KLK3, ENO1, SLC26A3*, and *TMPRSS2* in LNCaP, C4-2B, and MDA PCa 2b, but not to *KLK3* and *SLC26A3* in the Enz-resistant 22Rv1 (miR-99b-5p/Enz vs. NC, **Figure 6**). Drastically, the combination of miR-99b-5p and Enz exerted a synergistic effect to further decrease the occupancies of mTOR (or mTOR/AR) on these genes in all the tested PCa cell lines, including the Enz-resistant CRPC and AA PCa (miR-99b-5p/Enz vs. miR-99b-5p and miR-99b-5p/Enz vs. Enz, **Figure 6**). Taken together, these results suggested that miR-99b-5p and Enz effectively block the recruitment of mTOR (or mTOR/AR) to the target genes involved in the metabolic reprogramming in CRPC and/or PCa progression to CRPC. Furthermore, the combination of miR-99b-5p and Enz triggers a synergistic effect to significantly block the recruitment of mTOR and mTOR/AR onto their target genes, potentially serving as a novel therapeutic strategy for treating the resistant CRPC and aggressive AA PCa.

## Discussion

It has been evident that therapeutics mediating AR inhibition (using AR antagonist, such as Enz) are primarily effective but ultimately developing resistance to ADT development, which is an event termed CRPC. This development/progression to CRPC typically leads to an incurable disease (38). Therefore, a validation of important biomarkers is a crucial step for detecting and observing the progression of aggressive PCa to CRPC (39–41). There is an urgent need for developing both protein and gene-based biomarkers, and microRNAs are well considered as potential diagnostic and/or prognostic biomarkers (in tissue, blood, serum/plasma, and urine of PCa patients) for PCa progression to CRPC (42–45). In our recent study, we have highlighted the potential of developing reciprocal miR-99b-5p/nuclear mTOR (downregulated/upregulated) as a prognostic biomarker for aggressive AA PCa and other metastatic solid tumors. Furthermore, our study suggested that miR-99b-5p/mTOR/AR signaling axis may play crucial roles in promoting AA PCa aggressiveness and also involved in the development of CRPC. Additionally, miR-99b-5p overexpression drastically sensitized the aggressive AA PCa to docetaxel (17). In this study, we further explored the functional roles of miR-99b-5p in modulating mTOR/AR/SMARCD1 signaling axis in AA PCa aggressiveness and CRPC progression. According to TargetScan algorithm (https://www.targetscan.org/vert_80/), miR-99b-5p is predicted to target/regulate ~60 genes, including mTOR and SMARCD1. AR was recently confirmed as a direct target of miR-99b-5p by previous studies (17, 29). Also, nuclear AR and mTOR are transcriptional regulators for hundreds of genes in AA PCa and CRCP (30, 46). Taken together, miR-99b-5p potentially functions as a critical epigenomic driver for AA PCa aggressiveness and CRPC development/progression.

Enz has frequently been used as a first line therapeutic compound for CRPC. However, the drug resistance mechanisms underlying the advanced PCa (such as CRPC and aggressive AA PCa) still remains elusive. The Enz resistance observed in some CRPCs is considerably thought to occur via constant activation of AR, bypass of the AR mechanism, and development of AR independence (29, 47). In our study, we used a panel of PCa cell lines, including LNCaP (androgen-responsive metastatic EA PCa), C4-2B (CRPC developed from parental LNCaP) and 22Rv1 (Enz-resistant CRPC), and MDA PCa 2b (androgen-independent AA PCa) to study the functional impacts of miR-99b-5p/mTOR/AR/SMARCD1 signaling axis in PCa aggressiveness and drug resistance in EA and AA PCa. Previously, we identified that AR signaling pathway is upregulated in AA PCa vs. EA PCa, and a set of AR-target genes are overexpressed in AA PCa vs. EA PCa (48). Our integrative genomic analysis further identified miR-99b-5p/mTOR (down-/up-regulated) as a core miRNA-mRNA reciprocal pairing contributing to the upregulation of mTOR and VEGF signaling in AA PCa (5). Particularly, nuclear mTOR (and pmTOR) is enriched in AA PCa vs. EA PCa, and transfection of miR-99b-5p inhibits mTOR/AR expression and blocks the nuclear translocation of mTOR and AR (17). The AR-coactivator, SMARCD1, is critical for activating AR-target genes in nucleus for CRPC progression (33). In this study, we attempted to explore the functional roles of miR-99b-5p in modulating mTOR/AR/SMARCD1 signaling in MDA PCa 2b (an AA PCa cell model with similar features of CRPC C4-2B, that is androgen-independent but responds to Enz treatment) vs. LNCaP (EA PCa, androgen-dependent and Enz-sensitive PCa) and 22Rv1 (androgen-independent and Enz-resistant PCa). The enrichment of nuclear mTOR, AR and SMARCD1 in MDA PCa 2b has indicated a more aggressive tumor phenotype in AA PCa vs. EA PCa (i.e., LNCaP, an androgen-dependent EA PCa with much lower nuclear mTOR, AR, and SMARCD1). On the other hand, the enrichment of nuclear AR/AR-V7 and SMARCD1 in C4-2B and 22Rv1 (demonstrating similar aggressiveness to MDA PCa 2b) reflects the importance of AR/SMARCD1-signaling in CRPC (**Figure 2**). In either case, miR-99b-5p mimic inhibits tumor growth/viability and sensitizes Enz-induced cytotoxicity in AA PCa and EA CRPC (**Figure 5**). Further mapping of global mTOR and AR occupancies across genomes (i.e., using RNA-seq) of MDA PCa 2b vs. 22Rv1 and C4-2B may further identify the similarity/difference in metabolic rewiring and mTOR/AR/SMARCD1-mediated signaling in AA PCa vs. EA CRPC.

It has been suggested that the upregulation of the AKT/mTOR signaling pathway plays a key role on activating AR signaling cascade and promoting drug resistance in PCa cells (49). The constitutive activation of AR via mTOR upregulation represents a complex pathway for promoting aggressiveness and drug resistance in CRPC (50). In addition, the aggressiveness of PCa cells oftentimes demonstrate hyperactivation of mTOR signaling pathway (51), strongly implicating the involvement of mTOR-mediated signaling in aggressiveness and/or Enz-resistance in refractory CRPC. Previous study has also indicated that the exposure to the AR agonist R1881 activates AR and triggers the nuclear translocation of mTOR (30). Furthermore, the mTOR-dependent metabolic reprogramming has been demonstrated as a critical process for transcriptional regulating the metabolic gene expression profiles required for CRPC, even in the absence of androgens (30). For the inhibition AR-signaling, metastatic PCa patients are treated with ADT, that significantly extend overall survival of the patients (52). However, the ADT targeted patients eventually progress to castration-resistance, wherein tumor cells grow and metastasize even at castrated level of androgens (53). Earlier findings have shown that CRPC remains constant to AR signaling and such different mechanisms including AR upregulation and activated AR mutations (54). Based on such mechanism, an alternative *AR* splice variant has been known to cause reactivation of AR signaling in CRPC (54). Compared to full-length AR, ARAR-V7 spliced isoform has previously been shown to differentially regulate the downstream genes (25). AR-V7 can inhibit a specific set of tumor suppressor genes, thereby causing a castration-resistance phenotype in PCa (25). Also, AR-V7 variant lacks the ligand binding domain (LBD), and therefore is resistant to the AR antagonists (i.e. abiraterone and enzalutamide), which are the currently available therapy for CRPC (55). To solve this problem, drugs/compounds that causes the degradation of both full-length AR and AR-V7 are currently under development (56). Our current study has shown that miR-99b-5p potentially serves as a therapeutic molecule to simultaneously inhibit AR and AR-V7 in the PCa, evident from the significant reduction of AR in all cell lines and AR-V7 in 22Rv1 treated with miR-99b-5p or miR-99b-5p/Enz (**Figure 4**). Previous study also indicated that miR-99b-5p targets/inhibits SMARCD1, a coactivator of AR (32). SMARCD1 (BAF60a) has been known as a member of SWI/SNF family of proteins and it is well recognized to interact with the Ligand Binding Domain (LBD) of AR through its FXXFF motif in an androgen-dependent manner (31). In addition, it has been shown to bind with glucocorticoid receptor (GR) and provides the docking site for the chromatin remodeling BRG1 complex (57). SMARCD1 has been reported to be regulated by hepatocyte-specific miRNA miR-122 (58), and has been validated to also be targeted/inhibited by miR-99b-5p in this study (**Figure 4**). Consistent with the earlier report (32), we have shown/validated that miR-99b-5p negatively regulates SMARCD1 expression at the protein level (**Figure 4**).

## Conclusion

**Figure 7:**
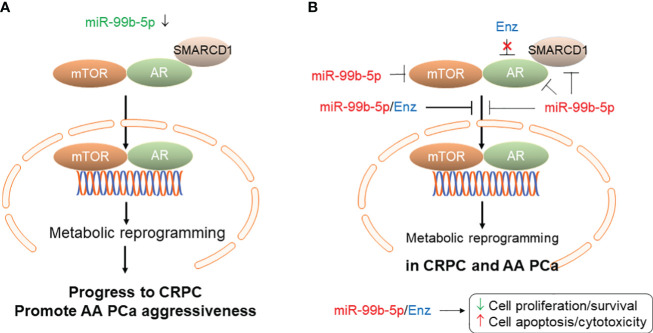
Proposed model of miR-99b-5p mediated mTOR/AR/SMARCD1 signaling axis involved in CRPC development/progression and/or AA PCa aggressiveness. **(A)** Downregulation of miR-99b-5p in PCa increases mTOR, AR and SMARCD1 protein levels and promotes the translocation of mTOR/AR/SMARCD1 complex to nucleus, consequently inducing metabolic reprogramming and triggering the development of CRPC or promoting the AA PCa aggressiveness. **(B)** Overexpression of miR-99b-5p as a therapeutic strategy for treating aggressive CRPC and AA PCa. Tumor suppressor miR-99b-5p inhibits protein expression of mTOR, AR and SMARCD1, blocks mTOR/AR/SMARCD1 translocation to nucleus and restores the metabolic gene program. Combining miR-99b-5p with Enz synergistically enhances the cell apoptosis and inhibits cell proliferation/survival in refractory CRPC and aggressive AA PCa.

In conclusion, our study has suggested that downregulation or deletion of miR-99b-5p could cause upregulation of mTOR, AR and AR coactivator SMARCD1, consequently activate a mTOR/AR-mediated metabolic gene reprogramming. This metabolic gene reprogram could further lead to the PCa aggressiveness and/or progression to CRPC disease. Taken together, the miR-99b-5p/mTOR/AR/SMARCD1 signaling axis may play important functional roles in promoting AA PCa aggressiveness and CRPC progression (as shown in **Figure 7A**). On the other hand, overexpression of miR-99b-5p (i.e. via transfection of miR-99b-5p mimic) targets/inhibits AR, mTOR and SMARCD1 simultaneously and blocks the translocation of mTOR/AR/SMARCD1 complex from cytoplasm to nucleus, consequently suppressing cell proliferation/survival and enhancing the cell apoptosis in PCa (especially AA PCA and CRPC). Furthermore, miR-99b-5p overexpression results in suppressing nuclear translocation of mTOR/AR/SMARCD1, thereby inhibiting mTOR/AR-mediated metabolic reprogramming and significantly sensitizes the CRPC and AA PCa to the Enz (or other AR antagonists) (**Figure 7B**). To date, this is the first study to investigate the functional roles of miR-99b-5p in the mTOR/AR/SMACR1 signaling axis in AA PCa and Enz-responding/resistant CRPCs. The synergistic inhibitory capacities of the miR-99b-5p/Enz combination may indicate a novel molecular strategy for targeting/treating the CRPC and aggressive AA PCa.

## Data availability statement

The original contributions presented in the study are included in the article/**Supplementary Material**. Further inquiries can be directed to the corresponding author.

## Author contributions

Conceptualization: B-DW. Methodology: MW, HG, and B-DW. Conducting of experiments and data acquisition: MW, HG, and B-DW. Data analysis and interpretation: MW and B-DW. Writing—original draft: MW and B-DW. Writing review and editing: MW and B-DW. Funding acquisition: B-DW. Resources: B-DW. Supervision: B-DW. All authors contributed to the article and approved the submitted version.
