# Supplementary material for: Tumor suppressive miR-99b-5p as an epigenomic regulator mediating mTOR/AR/SMARCD1 signaling axis in aggressive prostate cancer
Source: Front Oncol. 2023 Nov 7;13:1184186. doi: 10.3389/fonc.2023.1184186 (PMC10661933; doi:10.3389/fonc.2023.1184186)
Supplement: Supplementary file 1 [file DataSheet_1.pdf]

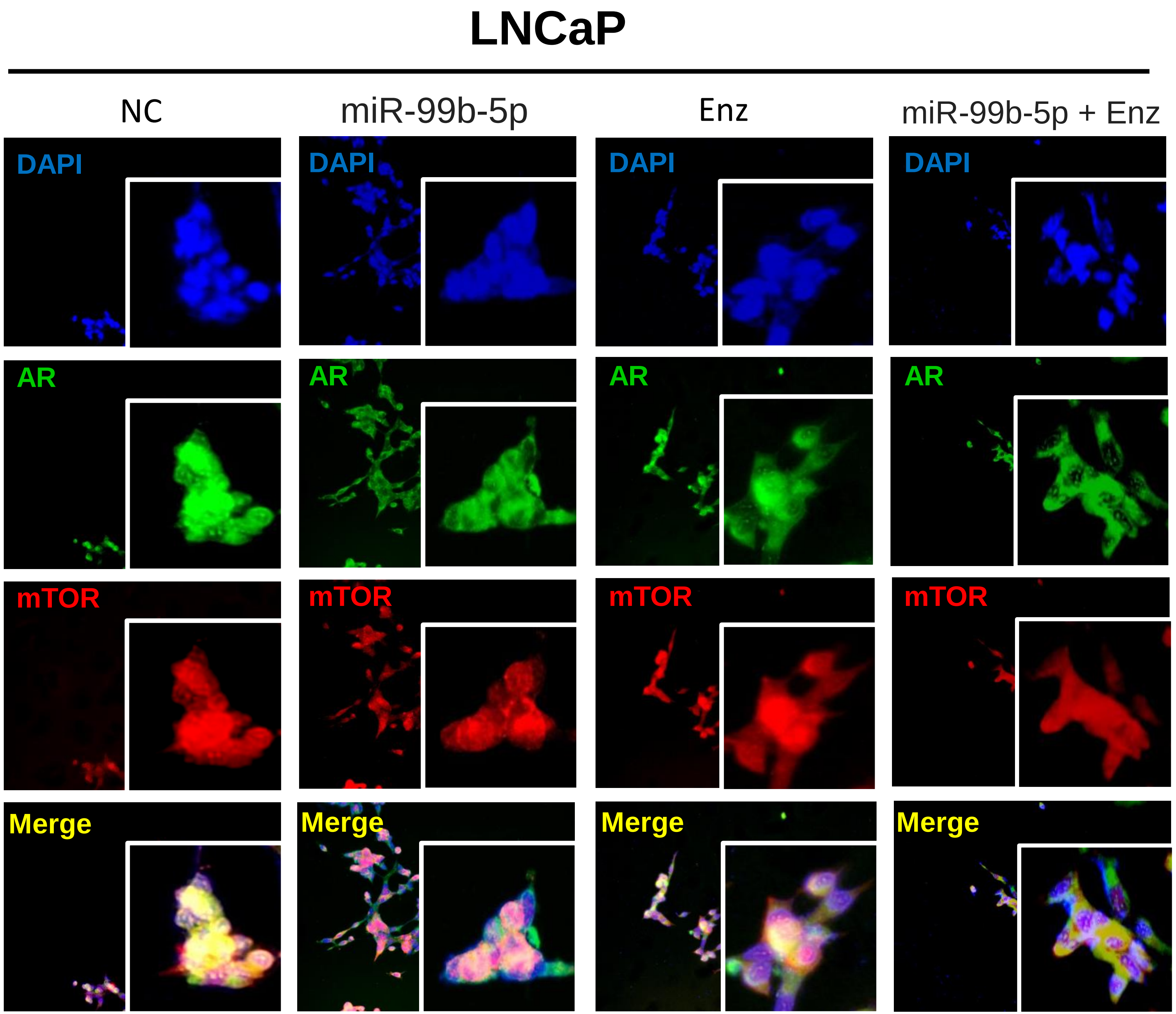

Supplementary Figure 1. Immunofluorescence staining revealed the cellular localizations and expression levels of AR and mTOR in LNCaP.

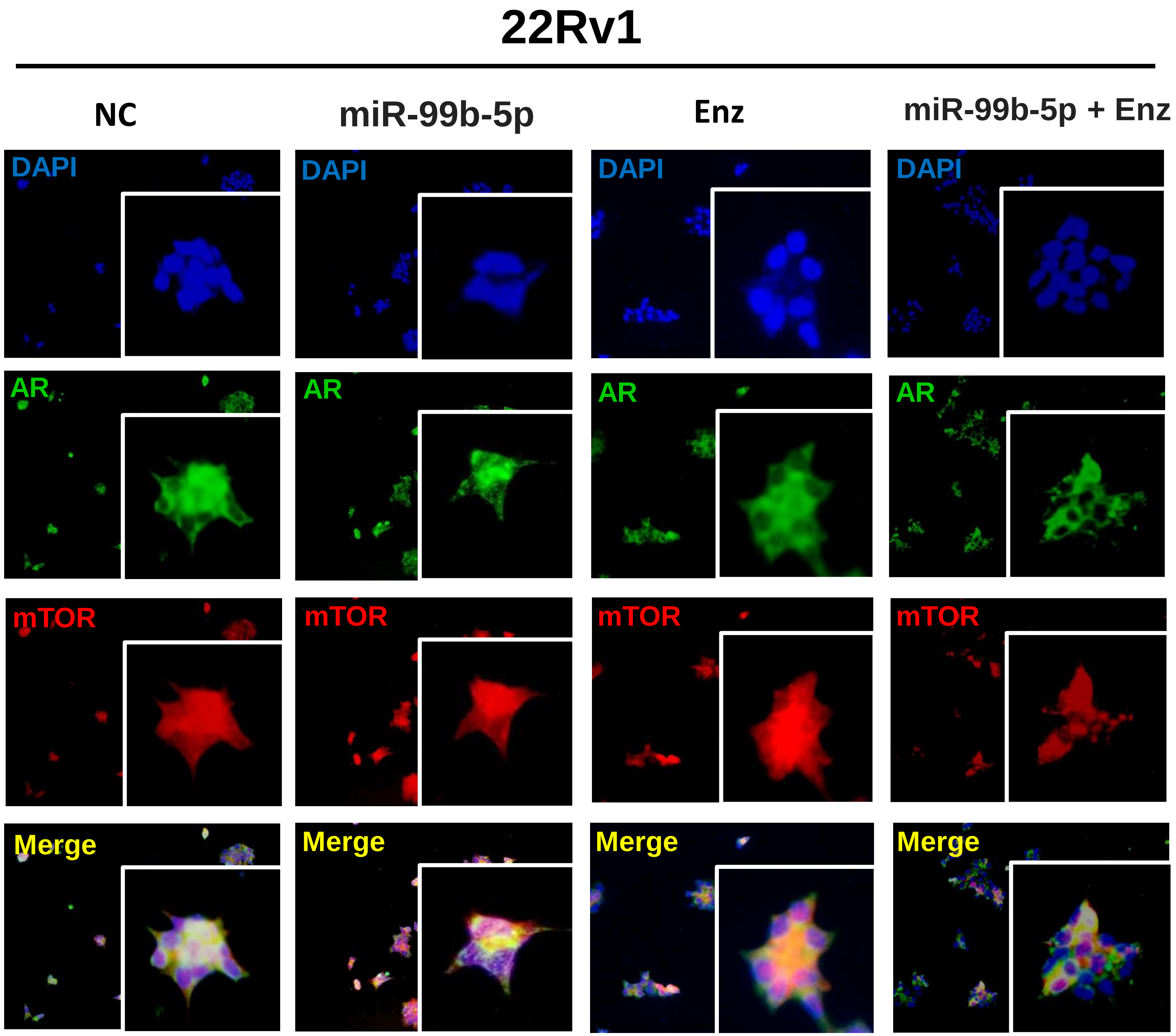

Supplementary Figure 2. Immunofluorescence staining revealed the cellular localizations and expression levels of AR and mTOR in 22Rv1.

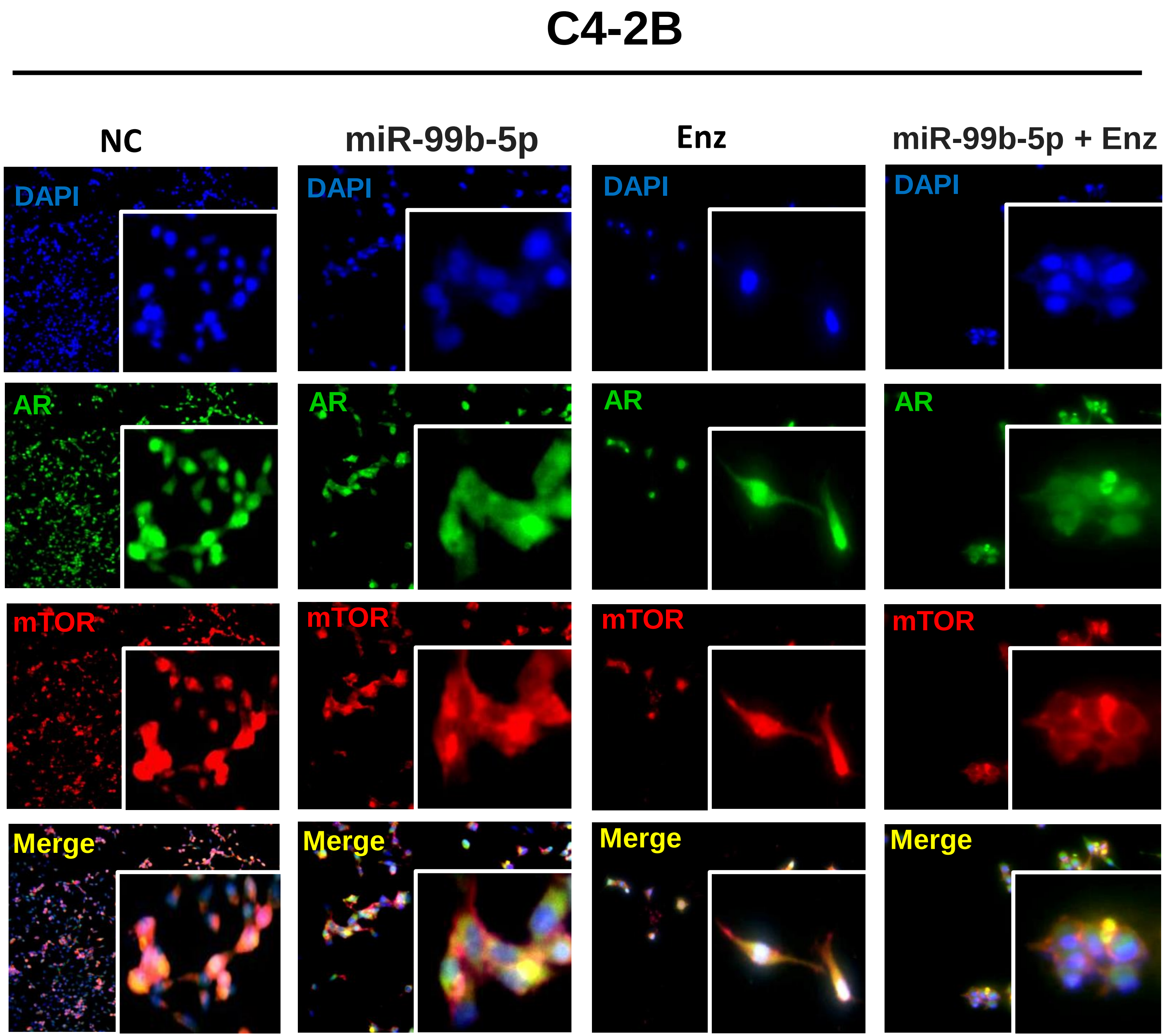

Supplementary Figure 3. Immunofluorescence staining revealed the cellular localizations and expression levels of AR and mTOR in C4-2B.

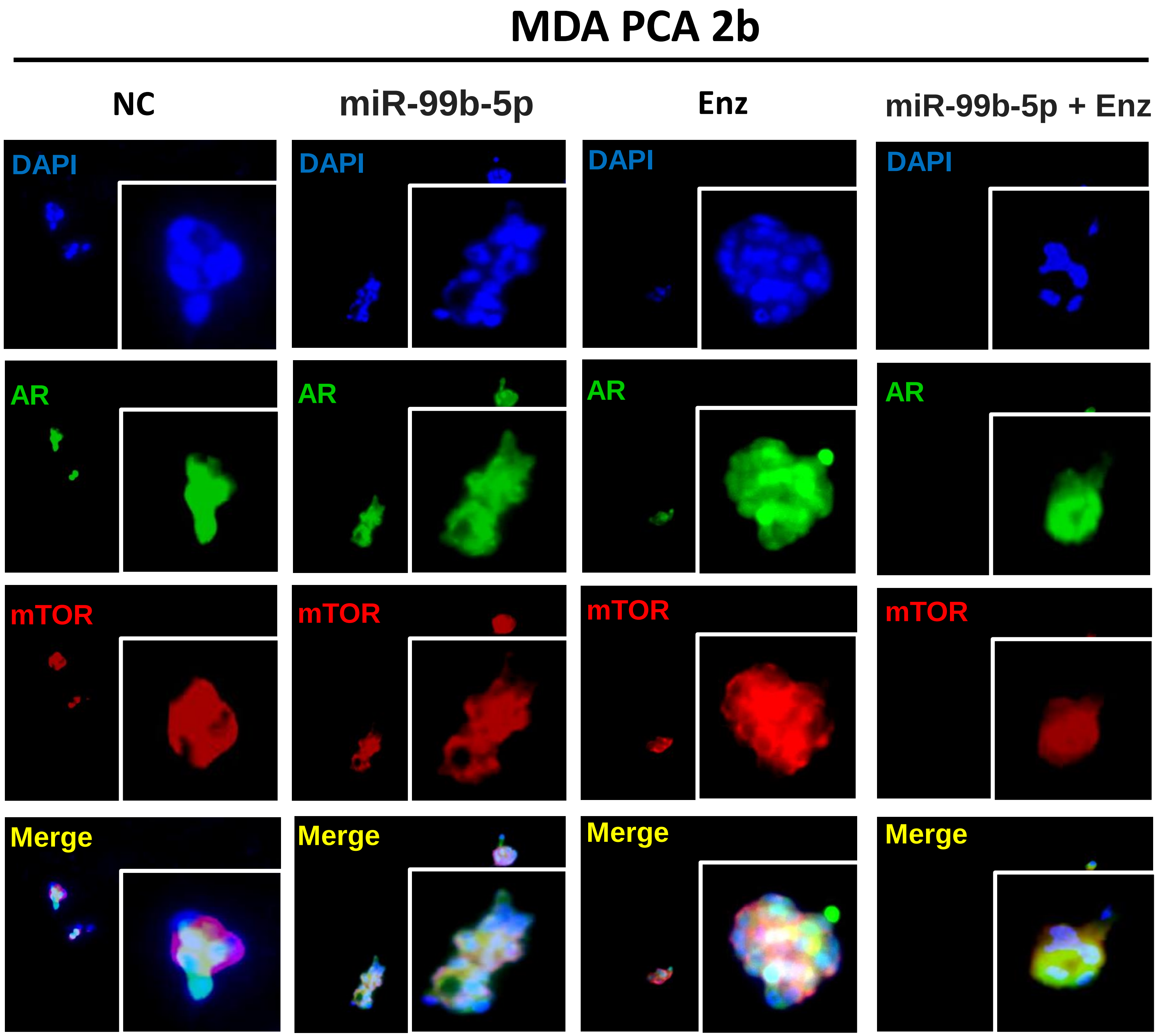

Supplementary figure 4. Immunofluorescence staining revealed the cellular localizations and expression levels of AR and mTOR in MDA PCa 2b.

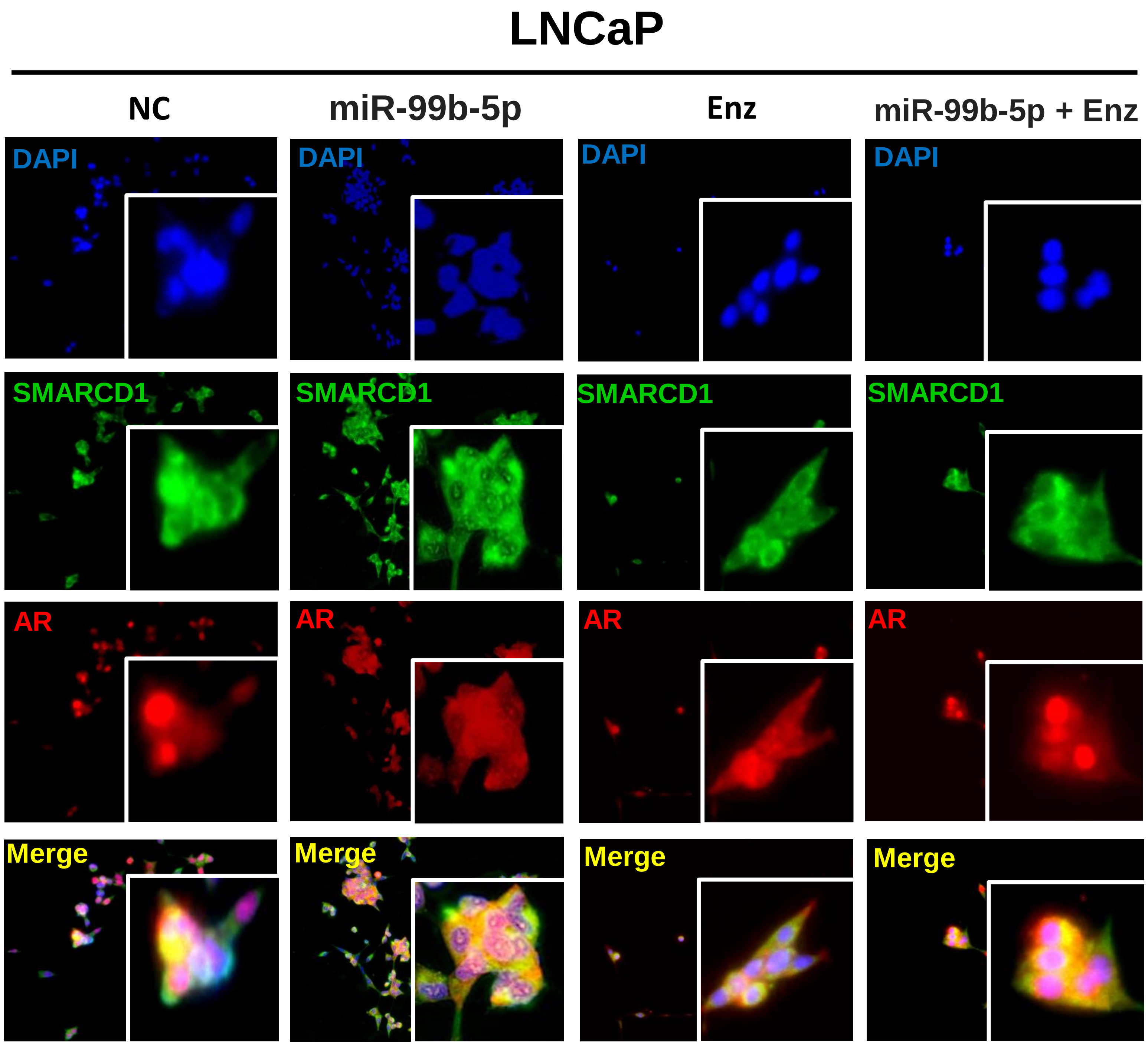

Supplementary Figure 5. Immunofluorescence staining revealed the cellular localizations and expression levels of AR and SMARCD1 in LNCaP.

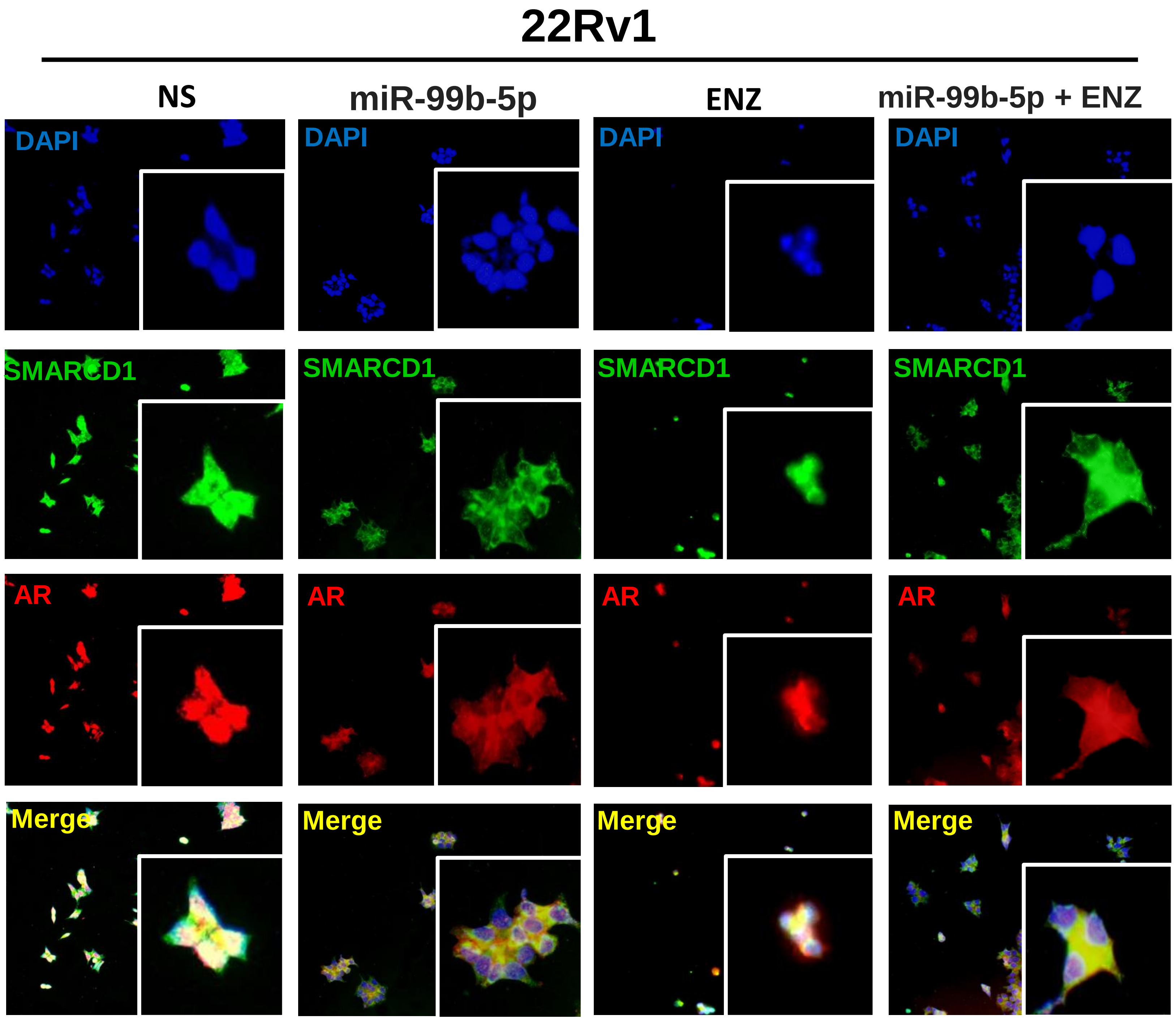

Supplementary Figure 6. Immunofluorescence staining revealed the cellular localizations and expression levels of AR and SMARCD1 in 22Rv1.

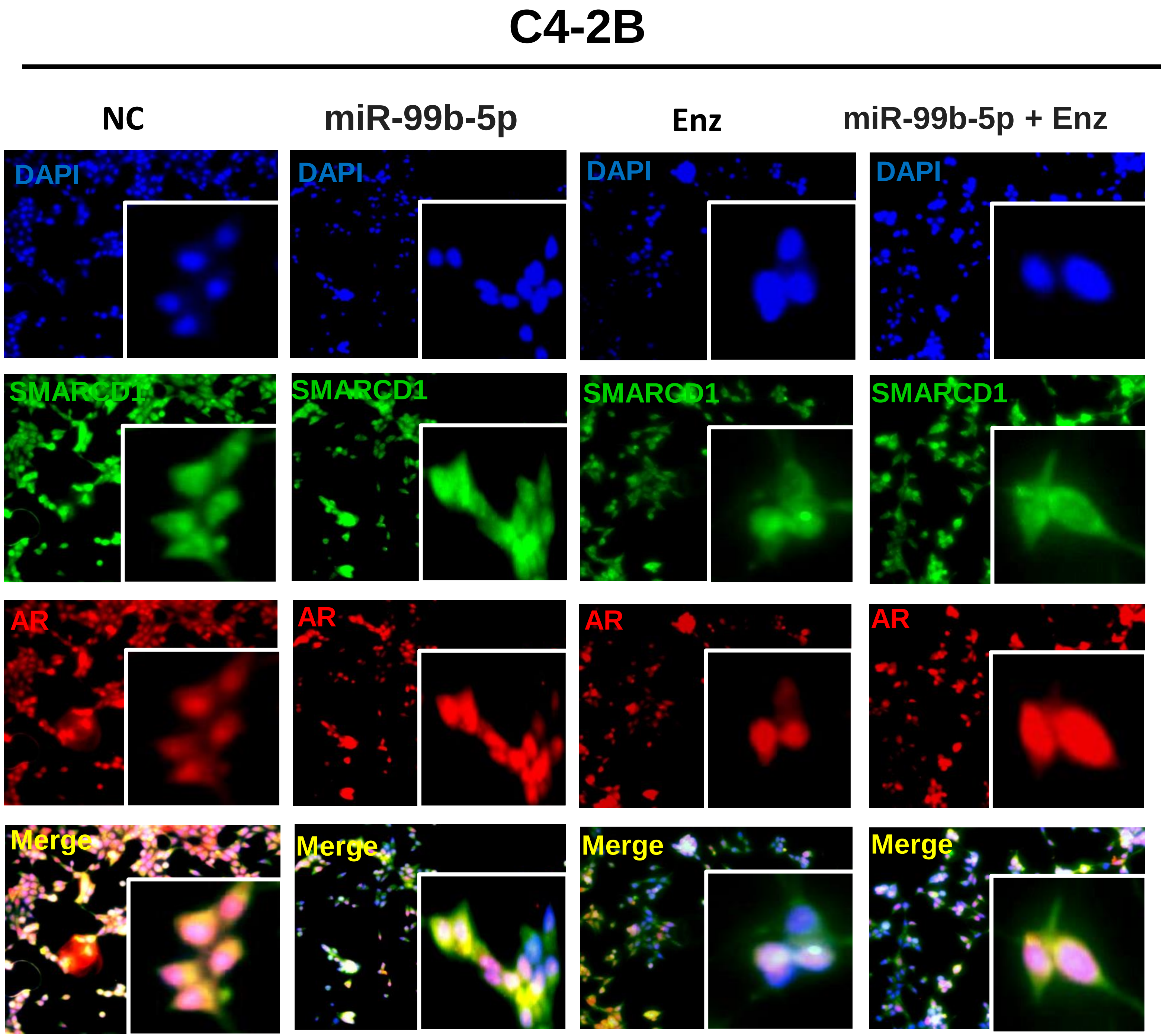

Supplementary Figure 7. Immunofluorescence staining revealed the cellular localizations and expression levels of AR and SMARCD1 in C4-2B.

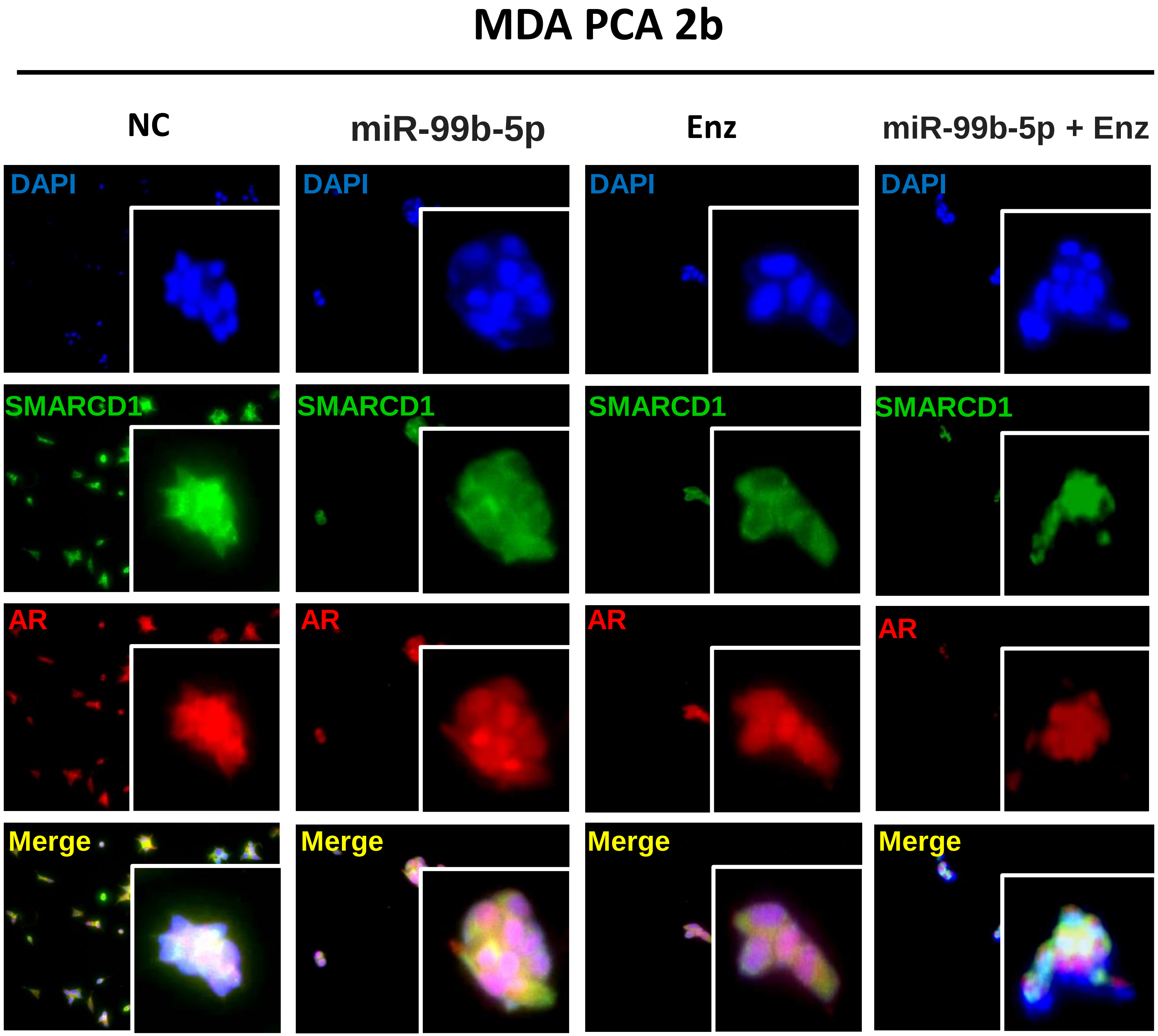

Supplementary Figure 8. Immunofluorescence staining revealed the cellular localizations and expression levels of AR and SMARCD1 in MDA PCa 2b.

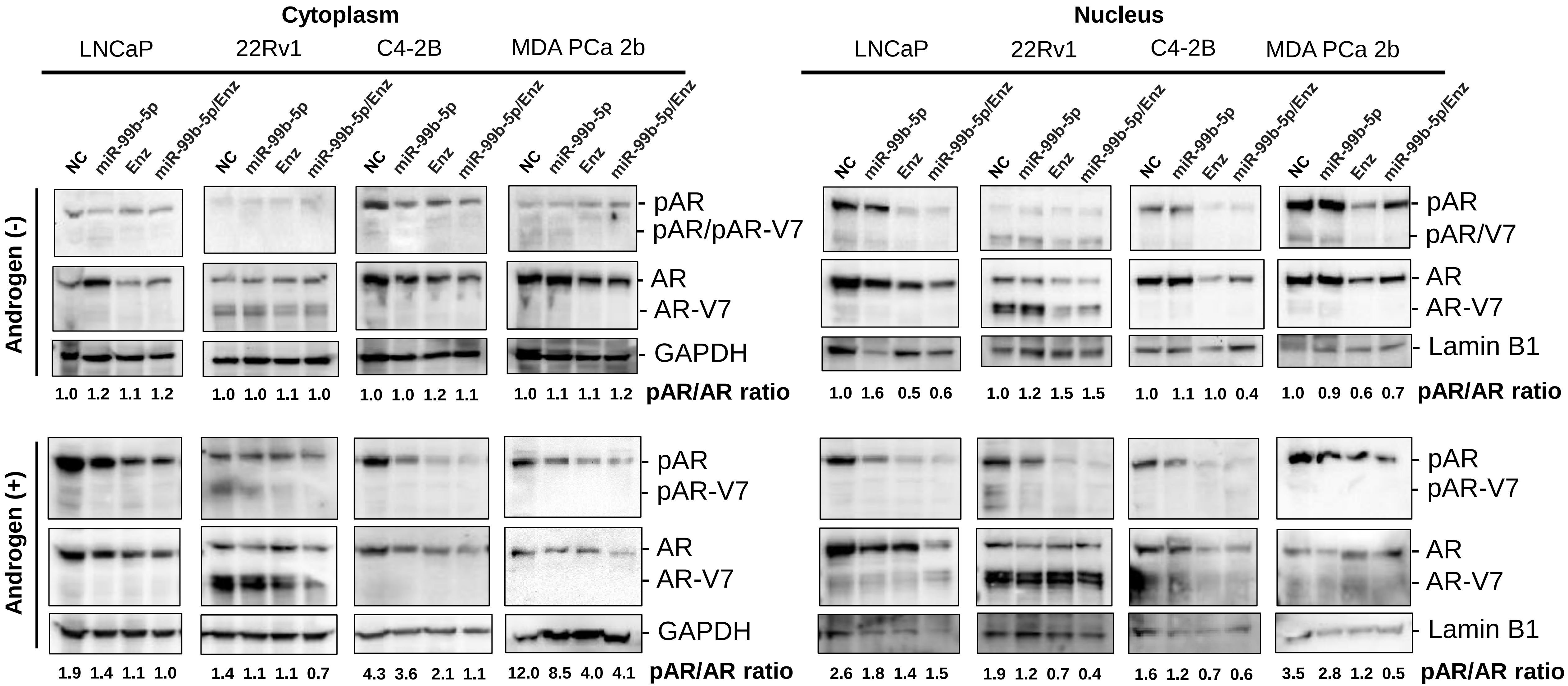

Supplementary Figure 9. Western blot analysis of pAR/pAR-V7 and AR/AR-V7 levels in LNCaP, 22Rv1, C4-2B and MDA PCa 2b under different treatments in the absence or presence of androgen. GAPDH and Lamin B1 are endogenous cytoplasmic and nuclear controls, respectively. NC: nonsense RNA/vehicle, miR-99b-5p: miR-99b-5p mimic/vehicle, Enz: nonsense RNA/enzalutamide, miR-99b-5p/Enz: miR-99b-5p mimic/enzalutamide. Cytoplasmic and nuclear pAR/AR ratios were calculated by using the equation of (pAR density/AR density)/GAPDH density and (pAR density/AR density)/Lamin B1 density, respectively. The pAR/AR ratio of NC treated cells in androgen (-) was defined as 1.0.

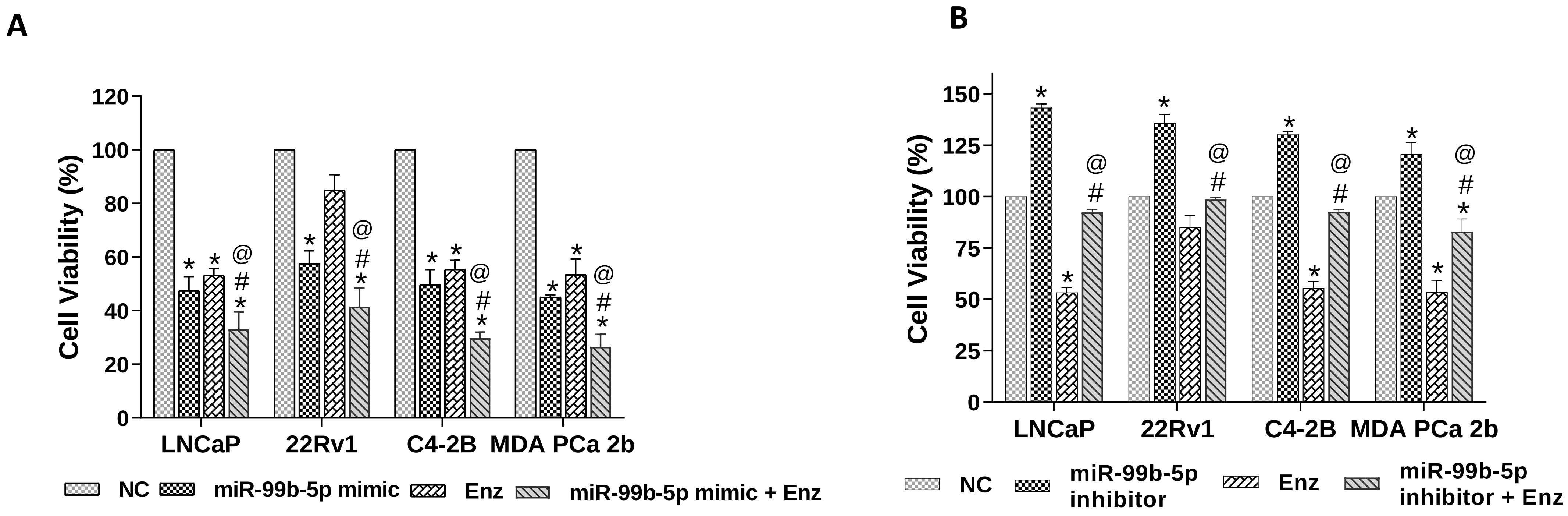

Supplementary Figure 10. Effects of NC, miR-99b-5p mimic, miR-99b-5p inhibitor, enzalutamide (Enz), and combination of miR-99b-5p mimic or inhibitor with Enz on cell viabilities of PCa cell lines. **(A)** MTT assays of the EA (LNCaP, 22Rv1 and C4-2B) and AA (MDA PCa 2b) PCa cells in response to NC, miR-99b-5p mimic, Enz or miR-99b-5p mimic/Enz treatments. **(B)** MTT assays of the EA (LNCaP, 22Rv1 and C4-2B) and AA (MDA PCa 2b) PCa cells in response to NC, miR-99b-5p inhibitor, Enz or miR-99b-5p inhibitor/Enz treatments. Significant difference on cell viabilities were recorded in miR-99b-5p mimic or inhibitor vs. NC, Enz vs. NC, or miR-99b-5p inhibitor/Enz vs. NC ($^{*}p$-value, < 0.05) and analyzed based on ANOVA with Dunnett's post-hoc test. Significantly different cell viability in miR-99b-5p mimic or inhibitor/Enz vs. miR-99b-5p mimic or inhibitor ($^{\#}p$-value < 0.05), and miR-99b-5p mimic or inhibitor/Enz vs. Enz ($^{@}p$-value< 0.05) were determined based on ANOVA with Tukey's post-hoc test. Each value was determined by the percentage of NC and each data point was represented as mean ± SD (n = 6).

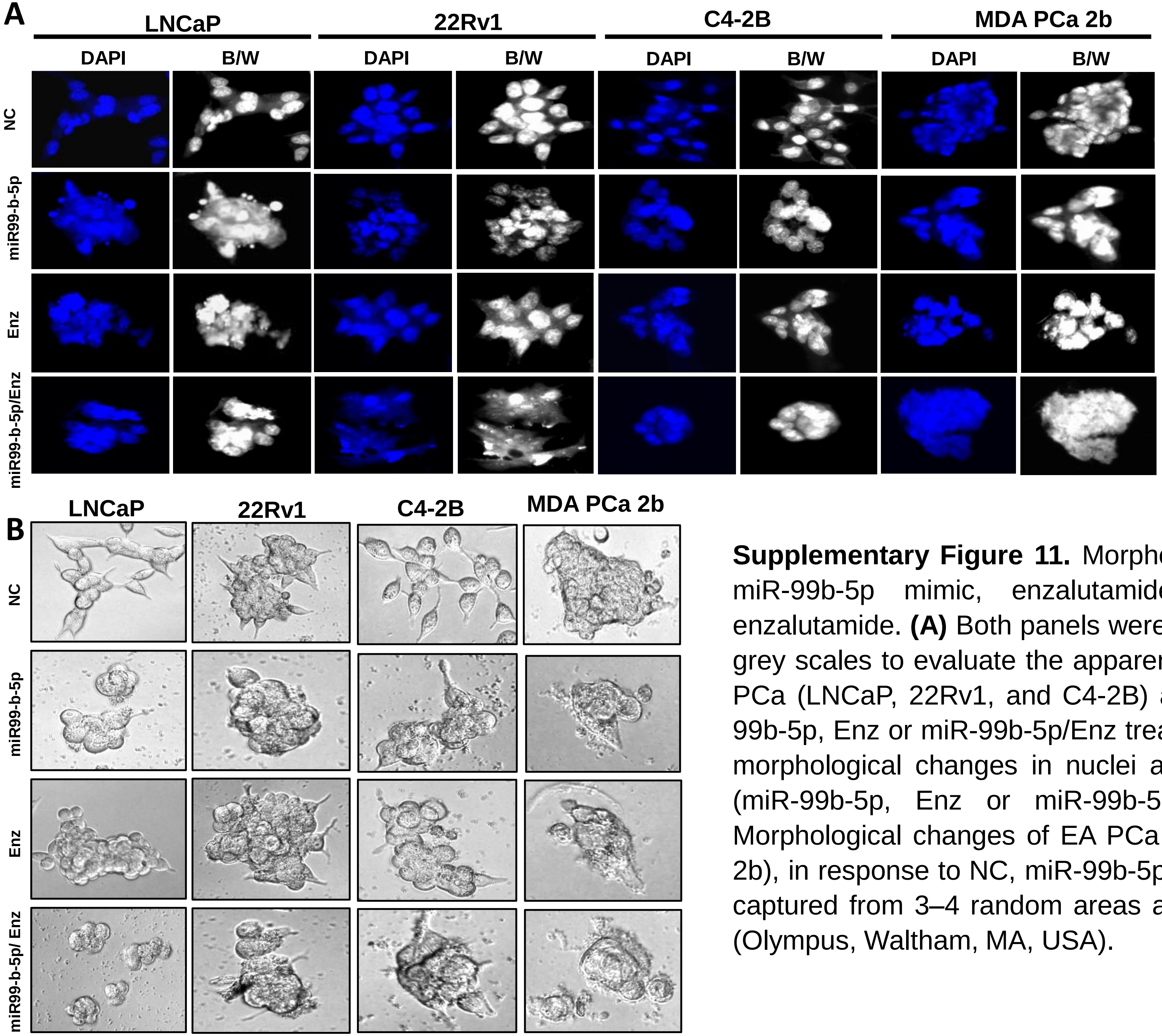

Supplementary Figure 11. Morphological changes in EA and AA PCa cells treated with miR-99b-5p mimic, enzalutamide, and combination of miR-99b-5p mimic with enzalutamide. **(A)** Both panels were acquired by representative DAPI signals with blue and grey scales to evaluate the apparent changes in shape and/or morphology of nuclei in EA PCa (LNCaP, 22Rv1, and C4-2B) and AA PCa (MDA PCa 2b), in response to NC, miR-99b-5p, Enz or miR-99b-5p/Enz treatments. Both (DAPI and grey-scaled) images show the morphological changes in nuclei as blebbing, invagination, or herniation under different (miR-99b-5p, Enz or miR-99b-5p/Enz) treatments, compared to NC groups. **(B)** Morphological changes of EA PCa (LNCaP, 22Rv1, and C4-2B) and AA PCa (MDA PCa 2b), in response to NC, miR-99b-5p, Enz or miR-99b-5p/Enz treatments. Cell images were captured from 3–4 random areas at 20× magnification by using CellSens V1.18 software (Olympus, Waltham, MA, USA).
